# Revealing ecotype influences on *Cistanche sinensis*: from the perspective of endophytes to metabolites characteristics

**DOI:** 10.3389/fmicb.2023.1154688

**Published:** 2023-06-27

**Authors:** Min Zhang, Yujing Miao, Xinke Zhang, Xiao Sun, Minhui Li, Linfang Huang

**Affiliations:** ^1^Inner Mongolia Key Laboratory of Characteristic Geoherbs Resources Protection and Utilization, College of Pharmacy, Baotou Medical College, Baotou, China; ^2^Key Laboratory of Chinese Medicine Resources Conservation, State Administration of Traditional Chinese Medicine of the People’s Republic of China, Institute of Medicinal Plant Development, Chinese Academy of Medical Sciences and Peking Union Medical College, Beijing, China; ^3^Inner Mongolia Hospital of Traditional Chinese Medicine, Hohhot, China; ^4^Inner Mongolia Traditional Chinese and Mongolian Medical Research Institute, Hohhot, China; ^5^State Key Laboratory of Southwestern Chinese Medicine Resources, Chengdu University of Traditional Chinese Medicine, Chengdu, China

**Keywords:** *Cistanche sinensis*, metabolome, microbiome, quality difference, potential correlation

## Abstract

**Introduction:**

Plant microorganism is critical to plant health, adaptability, and productive forces. Intriguingly, the metabolites and microorganisms can act upon each other in a plant. The union of metabolomics and microbiome may uncover the crucial connections of the plant to its microbiome. It has important benefits for the agricultural industry and human being health, particularly for Chinese medical science investigation.

**Methods:**

In this last 2 years study, on the strength of the UPLC–MS/MS detection platform, we accurately qualitatively, and quantitatively measured the *Cistanche sinensis* fleshy stems of two ecotypes. Thereafter, through high-throughput amplicon sequencing 16S/ITS sequences were procured.

**Results:**

PhGs metabolites including echinacoside, isoacteoside, and cistanoside A were significantly downregulated at two ecotypes of *C. sinensis*. Add up to 876 metabolites were monitored and 231 differential metabolites were analyzed. Further analysis of 34 core differential metabolites showed that 15 compounds with up-regulated belonged to phenolic acids, flavonoids, and organic acids, while 19 compounds with down-regulated belonged to phenolic acids, flavonoids, alkaloids, amino acids, lipids, and nucleotides. There was no noteworthy discrepancy in the endophytic bacteria’s *α* and *β* diversity between sandy and loam ecotypes. By comparison, the *α* and *β* diversity of endophytic fungi was notably distinct. The fungal community of the loam ecotype is more abundant than the sandy ecotype. However, there were few such differences in bacteria. Most abundant genera included typical endophytes such as *Phyllobacterium*, *Mycobacterium*, *Cistanche*, *Geosmithia,* and *Fusarium*. LEfSe results revealed there were 11 and 20 biomarkers of endophytic bacteria and fungi in *C. sinensis* at two ecotypes, respectively. The combination parsing of microflora and metabolites indicated noteworthy relativity between the endophytic fungal communities and metabolite output. Key correlation results that *Anseongella* was positive relation with Syringin, *Arsenicitalea* is negative relation with 7-methylxanthine and *Pseudogymnoascus* is completely positively correlated with nepetin-7-*O*-alloside.

**Discussion:**

The aim of this research is: (1) to explore firstly the influence of ecotype on *C. sinensis* from the perspective of endophytes and metabolites; (2) to investigate the relationship between endophytes and metabolites. This discovery advances our understanding of the interaction between endophytes and plants and provides a theoretical basis for cultivation of *C. sinensis* in future.

## 1. Introduction

*Cistanche sinensis* G. Beck is a unique species in both medicine and food in China, which is a genus of Cistanche in the Orobanchaceae family. Its stem is bright yellow, corolla tubular bell shape, light yellow, mainly distributed in Inner Mongolia, Gansu, and Ningxia provinces (Jiang and Tu, 2009). The current research of *C. sinensis* mainly focuses on the comparison of the characteristic map, the identification of its similar products, and the organelle genomics (Tu et al., 2007; Shi et al., 2009; Gao et al., 2019; Liu et al., 2019, 2020; Song et al., 2019; Miao et al., 2022). The similarity of HPLC characteristic maps between *Cistanche deserticola* and *C. sinensis* is only 0.053 (Jiang et al., 2009). Poliumoside and 2′-acetylpoliumoside (brandioside) are unique components in *C. sinensis*, but not detected in *C. deserticola* (Jiang et al., 2009). It has been covered (Tu et al., 2007; Jiang et al., 2009) that the chemical components of *C. sinensis* are mainly phenylethanoid glycosides, iridoid glycosides, and polysaccharides, among which phenylethanoid glycosides are the most important active components. Verbascoside has vasodilating-promoting activity (Yoshikawa et al., 2006), estrogen/anti-estrogen-like effect (Papoutsi et al., 2006), and anti-inflammation activity (Sahpaz et al., 2002), and poliumoside has antioxidative activity (He et al., 2000; Tai et al., 2009) and neuroprotective effect (Koo et al., 2005), which indicates that *C. sinensis* has certain medicinal development value to a certain extent.

In late years, microbiome and metabolomics have been given a lot of attention. The foregone investigation has bespoken that metabolite can mold the microbiome in plants (Jacoby et al., 2020, 2021). As is known to all, microorganisms exist in internal and external host plants, and hosts offer the interrelated nutrient substances for these microorganisms. The development circumstance of the plant decides what it can offer the microorganism. Astonishingly, the plant microbiome also can affect the relevant plant metabolome (Scherling et al., 2009; van de Mortel et al., 2012; Huang et al., 2014; Ryffel et al., 2016; Etalo et al., 2018; Huang et al., 2019). For instance, the contents of flavonoids and soil nitrogen in sugarcane stems were extremely relevant to the composition of soil microorganisms. Germs at the generic level showed biggish correlations with different metabolites, 6 genera all alone related to 90.9% of sugarcane metabolites, which play a primary metabolism part in sugarcane (Huang et al., 2021). Conjoint analysis of *Tricyrtis macropoda* microflora and metabolites revealed an outstanding relationship between the metabolite output and endophytic fungi. Variations in local leaf color may be caused by changes in these metabolites (Wang Y. et al., 2021). There were different alkaloid metabolites in *Aconitum vilmorinianum* roots from two producing areas, and the absorption of root microbiota was significantly different. Based on bioinformatics analysis, we identified the underlying bacteria and fungi affecting the alkaloid metabolome of *A. vilmorinianum* (Li et al., 2022).

*Cistanche sinensis* is usually born in sandy land, gravel land, or hilly slope in a desert steppe belt and desert area, located at an altitude of 1,000–2,240 meters. For one thing, *C. sinensis* plays a key part in improving the ecotope in drought regions. For another, *C. sinensis* adapts to different biotopes and comes into being an individual ecological type, which may produce unique chemical characteristics and microbial diversity. These characteristics will in turn define the quality of *C. sinensis* (Huang and Juan, 2012). The Pigeon Mountain site that the team went to is a site of ancient human culture, dating from 1.27 to 0.8 thousand years ago, sparsely populated and mainly gravel land. According to the observation, there is a strong contrast between this place and Dawan Road, Xinglong Town, Tongxin County, Wuzhong City, and there are obvious differences in the appearance and morphology of *C. sinensis*, which leads us to explore the internal differences of *C. sinensis* in these two ecotypes.

In addition, the beneficial endophytes can maintain the growth health of plants and produce ideal secondary metabolites, which had important medicinal and economic value (Trivedi et al., 2020). *Cistanche sinensis* is now included in near threatened (NT) on the *IUCN Red List of Threatened Species*. For the first time, our research goal is to explore the correlation between endophytes and metabolites of *C. sinensis* from the perspective of protecting raw material supply and wild resources, to offer academic direction to the effective cultivation of *C. sinensis* in the future and to solve the resource shortage.

Our research first time inquired two major questions: (1) What are the biomarkers that distinguish endophytes and metabolites of the two ecotypes of *C. sinensis*? (2) Are microorganisms in plants related to changes in metabolites? To solve these problems, we studied the composition, diversity, and function prediction of endophytic microbial communities, as well as the relative content and classes of metabolites of *Cistanche cistanche*’s two ecotypes. And we analyzed the correlation between microbiome and metabolite.

## 2. Materials and methods

### 2.1. Sample collection and processing

**Figure 1:**
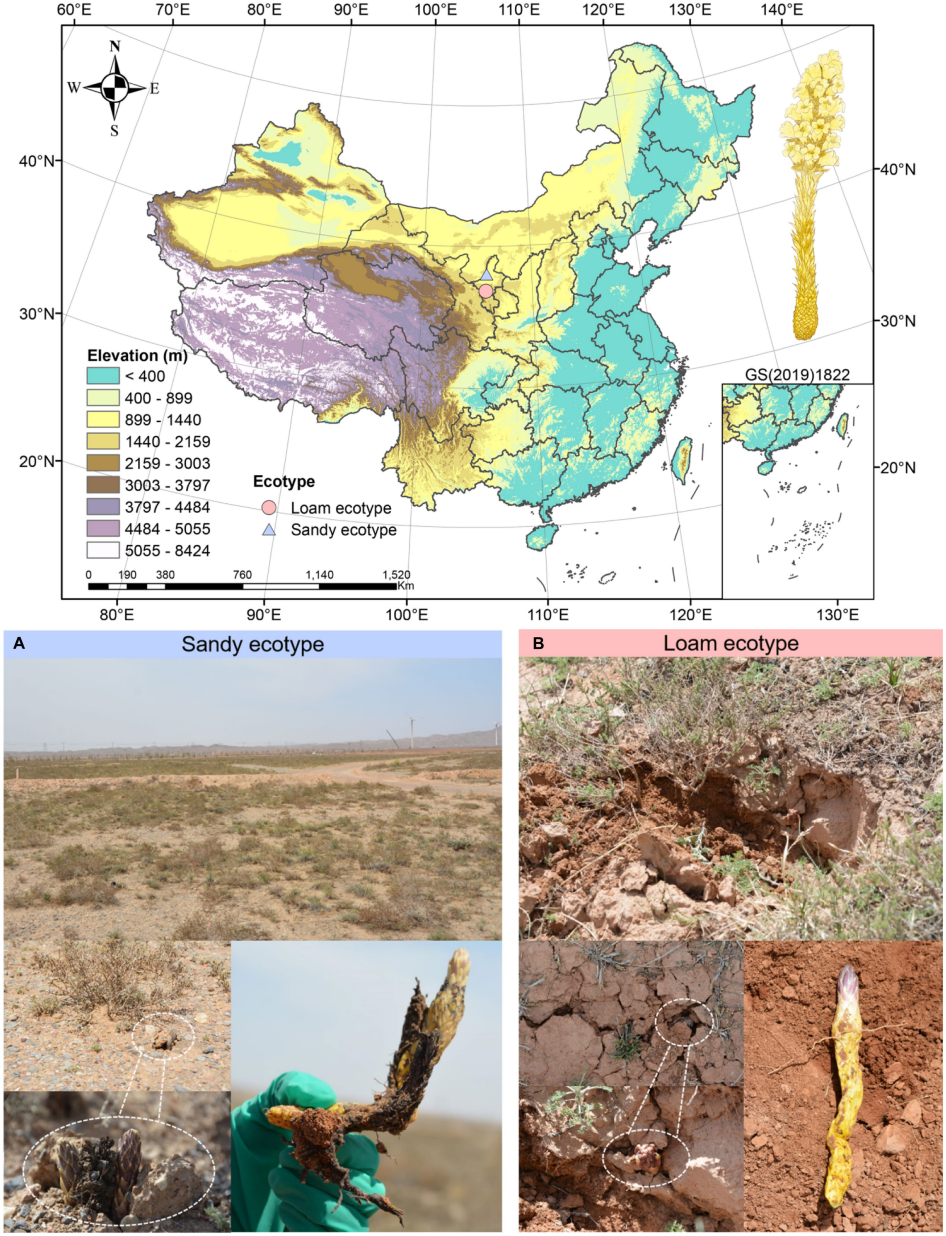
Field environment of *Cistanche sinensis* under two ecotypes. **(A)** Qingtongxia County (38.0556°N, 105.842°E, 1210 m), Yinchuan City. **(B)** Tongxin County (36.876°N, 105.781°E, 1570 m), Wuzhong City.

In April 2021, we gathered *C. sinensis* in Qingtongxia County (38.0556°N, 105.842°E, 1210 m), Yinchuan City, and Tongxin County (36.876°N, 105.781°E, 1570 m), Wuzhong City, respectively, in northwest China’s Ningxia Hui Autonomous Region (Figure 1). Below, Qingtongxia is referred to as GCS, and Tongxin is referred to as HCS. According to the soil mechanical composition, GCS and HCS were divided into sandy and loam ecotypes (Supplementary Table 1). We collected the fleshy stems of *C. sinensis*, and collected 6 biological duplicates for each site, totaling twelve samples. All the samples collected were healthy plants. Twelve *C. sinensis* fleshy stems were split into two groups for management, respectively. The first group was to clean the dust on the surface with sterile water, in liquid nitrogen for 30 s, for the extraction of metabolites. Tnhe second group was to remove microorganisms from the surface of the sample, soak it in 75% alcohol for 2 min, then soak it for 3 min in 5% hypochlorite, and ultimately wash it in sterile water 3 times. The samples were frozen and sterilized in liquid nitrogen for the 30s, and the DNA extraction kit extracted the total DNA. An ultra-low temperature refrigerator at −80°C preserved all and backup samples for follow-up experiments. Chinese Academy of Medical Sciences and Peking Union Medical College Institute of Medicinal Plant Development Professor Huang Linfang officially identified plant materials.

### 2.2. Determination of total antioxidant capacity *in vitro*

#### 2.2.1. Sample preparation

According to the tissue mass (*g*): the volume of extraction liquid (mL) was 1:5–10 (about 0.1 g tissue was weighed and 1 mL of extraction liquid was added), ice bath homogenization was carried out, then 10,000 g tissue was centrifuged at 4°C for 10 min, the supernatant was taken. The supernatant was successively diluted to 10, 20, 30, 40, 50, 60, 70, 80, and 90 mg/mL and placed on ice for testing. Biology was repeated 3 times for each group of samples. The ABTS, DPPH, and FRAP kits used in this study were purchased from Suzhou Keming Biotechnology Co., LTD (Wang et al., 2022).

#### 2.2.2. ABTS assay

About the kit instructions, preheat the enzyme-labeled instrument for 30 min and adjust the wavelength to 734 nm. The first preparation of the working liquid is to take one bottle of reagent 2, add 11 mL of reagent 1, shake and mix for 20 min, then let it stand, and take the supernatant for use (ready to use). In the 96-well plate, the blank tube is added with an extraction solution of 10 μL and a working solution of 190 μL. The measuring tube is to add different concentrations of samples 10 μL and 190 μL working liquid. After full mixing, the absorption value was measured within 10 min. The total antioxidant capacity (ABTS assay, micro method 100 T/96S) is calculated as follows:

Total antioxidant capacity (μmol Trolox/g fresh weight) = 1.424*(△A + 0.0012)/W


$$\left(\Delta A=A_{\mathrm{blank}}-A_{\mathrm{measurement}}\mathrm{;W}:\mathrm{sample quality;g}\right)$$

#### 2.2.3. DPPH assay

About the kit instructions, preheat the enzyme-labeled instrument for 30 min and adjust the wavelength to 515 nm. In the 96-well plate, the blank tube was added with extraction solution 10 μL and reagent 1,190 μL; The measuring tube is added with a sample of different concentrations of 10 μL and a reagent of 190 μL. After full mixing, the light absorption value was measured within 20 min after the reaction at room temperature. The total antioxidant capacity (DPPH assay, micro method 100 T/96S) is calculated as follows:


$$\begin{array}{c} \mathrm{Total antioxidant capacity}\\ \;\left(\mu \mathrm{mol Trolox}/g\;\mathrm{fresh weight}\right) \end{array}=1.414^{*}\left(\Delta A+0.0081\right)/W$$

$$\left(\Delta A=A_{\mathrm{blank}}-A_{\mathrm{measurement}}\mathrm{;W}:\mathrm{sample quality;g}\right)$$

#### 2.2.4. FRAP assay

About the kit instructions, preheat the enzyme-labeled instrument for 30 min and adjust the wavelength to 593 nm. The first preparation of the mixture is to mix reagent 1, reagent 2, and reagent 3 at a ratio of 10:1:1, and pre-temperature at 37°C before use (ready for use). In the 96-well plate, the blank tube is the mixture of extract 10 μL and 190 μL; The measuring tube is mixed with samples of different concentrations of 10 μL and 190 μL. After full mixing, the absorption value was determined within 20 min of the reaction. The total antioxidant capacity (FRAP assay, micro method 100 T/96S) is calculated as follows:


$$\begin{array}{c} \mathrm{Total antioxidant capacity}\\ \left(\mu \mathrm{mol Trolox}/g\;\mathrm{fresh weight}\right) \end{array}\;=0.8054^{*}\left(\Delta A-0.0134\right)/W$$

$$\left(\Delta A=A_{\mathrm{measurement}}-A_{\mathrm{blank}}\mathrm{;W}:\mathrm{sample quality;g}\right)$$

### 2.3. Metabolite extraction and analysis

Freeze-dried and ground sample into powder. 1.2 mL 70% methanol dissolved 100 mg powder and extracted by the vortex. Vortex 6 times, vortices every 30 min, each lasting 30 s. The evenly mixed extracts were refrigerated at 4°C overnight and centrifuged at 4°C for 10 min at 12000 RPM. After centrifugation, 0.22 μm microporous filter membrane filtered supernatant and measured. The filtrate was studied by ultra–high-performance liquid chromatography–tandem mass spectrometry (UPLC–MS/MS), and the medium supernatant of all samples was mixed to make quality control (QC) samples (Doppler et al., 2016) for metabolomics analysis.

UPLC (SHIMADZU Nexera X2) and MS/MS (Applied Biosystems 4,500 QTRAP) primarily contained the sample data acquisition instrument system. Liquid phase conditions mainly include the Agilent SB-C18 column; Mobile phase A is ultra-pure water with 0.1% formic acid, and mobile phase B is acetonitrile with 0.1% formic acid. The elution gradient is 5% of phase B at 0.00 min, 95% of phase B at 0.00–9.00 min, 95% of phase B at 9.00–10.00 min, 5% of phase B at 10.00–11.10 min, and 5% of phase B at 11.10–14.00 min. The column temperature was 40°C. The flow rate was 0.35 mL/min. The sample size was 4 μL. The main conditions for mass spectrometry include ESI as an ion source, turbo spray, source temperature 550°C, ion spray voltage (IS) as negative ion mode − 4,500 V, positive ion mode 5,500 V, curtain gas (CUR), ion source gas I (GSI) and gas II (GSII) set at 25, 50, and 60 psi, respectively. The instrument was tuned and calibrated with 10 and 100 μmol/L propylene glycol solution in QQQ and LIT modes, respectively. The QQQ scan uses MRM mode and has the collision gas (nitrogen) set to medium. The collision-induced ionization parameter is set to high.

### 2.4. DNA extraction, sequencing, and processing

After genomic DNA extraction from the fleshy stems of *C. sinensis* at the sandy and loam ecotypes, the extracted genomic DNA was detected by 1% agarose gel electrophoresis. The internal transcribed spacer regions of the fungal ITS1 ribosomal RNA gene were amplified by PCR using the primers ITS1-1F-F CTTGGTCATTTAGAGGAAGTAA and ITS1-1F-R GCTGCGTTCTTCATCGATGC. The bacterial V3-V4 hypervariable region of 16S ribosomal RNA genes was amplified by PCR using the primers 338F-ACTCCTACGGGAGGCAGCAG and 806R-GGACTACHVGGGTWTCTAAT. The PCR was implemented on a Mastercycler Gradient (Eppendorf, Germany) employing 25 uL reaction volumes, involving 12.5 μL 2× Taq PCR MasterMix (Vazyme Biotech Co., Ltd., China), 3 μL BSA(2 ng/ul), 1 μL Reverse Primer(5 μM), 1 μL Forward Primer(5 μM), 7.47 μL ddH_2_O and 30 μL template DNA. for 45 s with a final extension at 72C for 10 min. The PCR products were purified employing an Agencourt AMPure XP Kit (Beckman Coulter, Inc., United States). Deep sequencing was enforced on Illumina Miseq/Novaseq (Illumina, Inc., United States) platform at Beijing Allwegene Technology Co., Ltd.

According to the barcode sequence, the different samples are uncoupled from the original data by QIIME (Caporaso et al., 2010) (v1.8.0) software. Raw data were filtered and spliced through Pear (Zhang et al., 2014) (v0.9.6) software. Consider deleting sequences shorter than 120 bp, with a low-quality score (≤20), and containing ambiguous bases. During splicing, the minimum overlap setting was 10 bp, and the mass ratio was 0.1. The chimera sequences of bacteria and fungi were wiped off employing Vsearch (v2.7.1) (Rognes et al., 2016) software after splicing. For bacteria, sequences less than 230 bp in length were removed and compared by the uchime (Edgar et al., 2011) method based on Gold Database. For fungi, sequences less than 120 bp in length were wiped off and compared employing the uchime method according to Unite database. Qualified sequences were gathered into operational taxonomic units (OTUs) at a similarity threshold of 97% dusing the Uparse (Edgar, 2013) algorithm of Vsearch (v2.7.1) software. To ensure that the coverage of all samples is fairly high, the data volume of all samples is uniform. The OTU representative sequences were compared with the BLAST (Ye et al., 2006) algorithm using Silva138 (Pruesse et al., 2007) and Unite 8.2 (Abarenkov et al., 2010) databases, respectively. The e-value threshold was set to 1e-5 to obtain the species classification information corresponding to each OTU and annotate the bacterial and fungal communities. To expurgate the items of chloroplasts, mitochondria, protoplasts, and plant, and percolate the unclassified pollutant sequences. The fleshy stem is sandy and loam ecotypes were isolated to evaluate the disparities after analyzing the unabridged dataset.

### 2.5. Statistical analysis of metabolome and microbiome

Samples’ metabolites were analyzed qualitatively and quantitatively on the strength of MWDB (Metware Biotechnology Co., Ltd. Wuhan, China). The principal component analysis (PCA) was performed by the statistics function prcomp within R (www.r-project.org). The hierarchical cluster analysis (HCA) results of metabolites emerged in the form of the tree heat map. HCA was carried out by R package ComplexHeatmap. The normalized semaphore intensity of metabolites in HCA (unit variance scaling) is visualized as a color spectrum. Notably regulated metabolites were resolved by VIP ≥ 1 and absolute log_2_FC (fold change) ≥ 1 between groups. VIP values were extracted from the results of orthogonal partial least squares–discriminant analysis (OPLS-DA) using R-package MetaboAnalystR, which also included score and permutation plots. Before OPLS-DA, the datum was log_2_ and mean centering. To refrain from over-fitting, a permutation test (200 permutations) was implemented.

Statistical analyses were performed employing R 4.2.0., QIIME (v 1.8.0), and Phython (v 2.7). Based on OTU and its abundance results, The alpha diversity index and beta diversity distance matrix were computed by QIIME software respectively, and R software was used to map the PCoA analysis. In addition, R software was used to analyze the bar graph according to the results of classification labeling and relative abundance. Use Python (v2.7) software for LEfSe analysis (Sun et al., 2020b,c).

### 2.6. Integrative analysis of the metabolome and microbiome

The LEfSe method was brought to analyze biomarkers with statistical discrepancies. The Spearman algorithm was brought to analyze the relevance between fungi and bacteria screened at the genus level and identified core differential metabolites, and the results were represented by a heat map. The results with a *p*-value of less than 0.05 and a correlation of more than 0.8 should be more clearly and obviously visualized by Cytoscape software (Sun et al., 2020a; Miao et al., 2023).

## 3. Results

### 3.1. Determination of total antioxidant capacity

**Figure 2:**
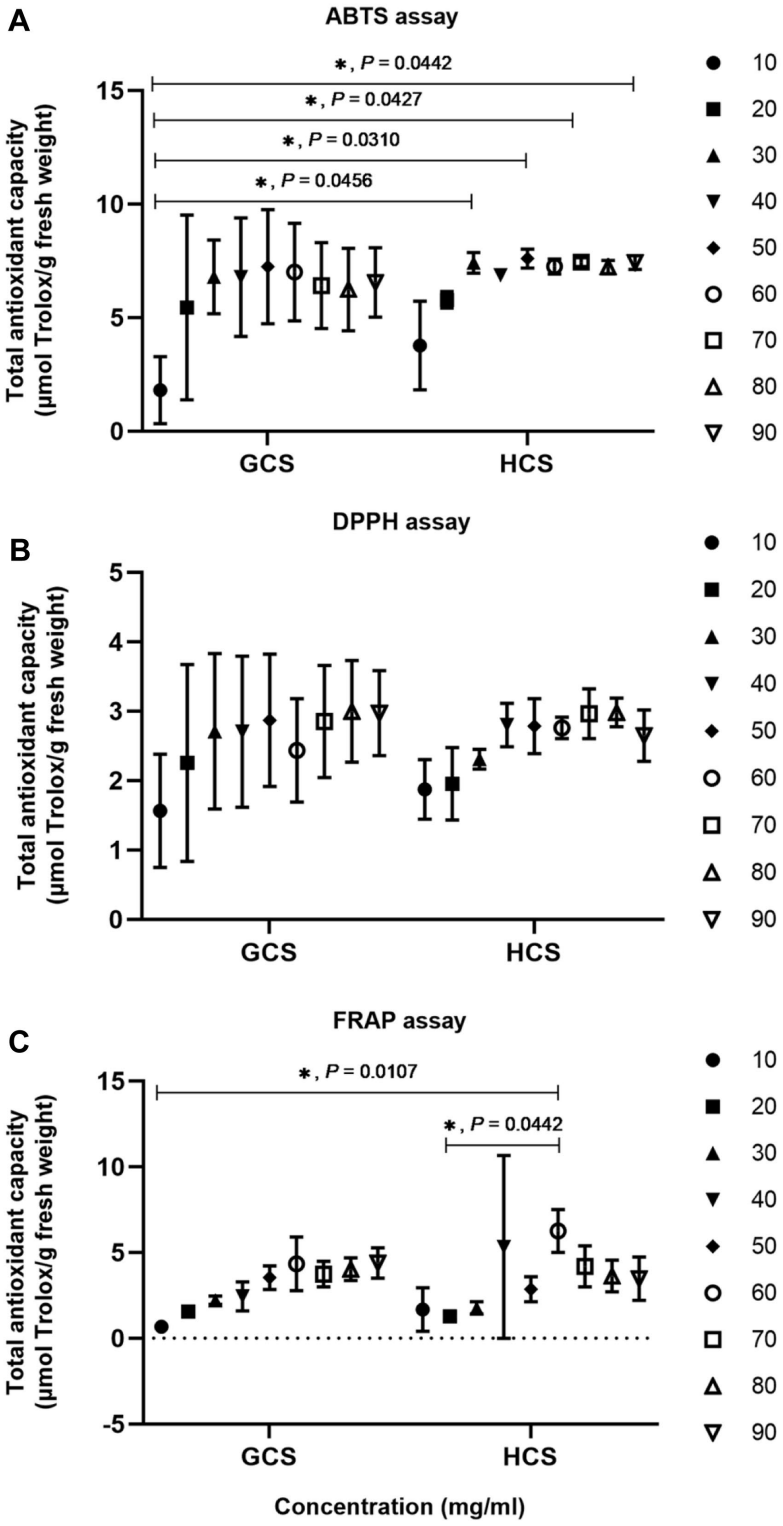
The antioxidant capacity of *Cistanche* sinensis at two ecotypes was analyzed by the two-way ANOVA method. “*” on behalf of “*p* <0.05” and if it is not marked significant, it is not significant. **(A)** ABTS assay. **(B)** DPPH assay. **(C)** FRAP assay.

We used ABTS, DPPH, and FRAP assay methods to evaluate the antioxidant activity of *Cistanche sinensis* at two ecotypes. On the whole, the total antioxidant capacity of *C. sinensis* with the loam ecotype is stronger than that with the sandy ecotype. The total antioxidant capacity of ABTS is shown in Figure 2A. When the mass concentration is 10-50 mg/mL, the total antioxidant capacity of *C. sinensis* at two ecotypes increases with the increase of sample concentration, and when the mass concentration is 60–90 mg/mL, the total antioxidant capacity of the sample tends to be level, indicating that the total antioxidant capacity reaches the maximum. The total antioxidant capacity of DPPH is shown in Figure 2B. Overall, the total antioxidant capacity of *C. sinensis* increases slowly when the mass concentration is 10–80 mg/mL. Interestingly, the total antioxidant capacity of the samples decreased at 60 and 90 mg/mL. The results showed that the total antioxidant capacity reached the maximum at 80 mg/mL. The total antioxidant capacity of FRAP was shown in Figure 2C, and the total antioxidant capacity of *C. sinensis* showed an increasing trend. However, when the mass concentration was 60 mg/mL, the total antioxidant capacity of the samples was the highest. The total antioxidant capacity of the samples reached the level of 70–90 mg/mL.

### 3.2. Metabolomic changes in *Cistanche sinensis* fleshy stems between Qingtongxia and Tongxin

To tell the difference between sandy and loam ecotypes in medicinal compositions in the Ningxia Hui Autonomous Region of northwest China, we performed metabonomics studies on the samples.

Before the difference analysis, PCA was carried out on the group samples for difference comparison to look into the change between the groups and among the samples within the groups (Supplementary Figure 1). The explanation by variable X (PC1) was 30.11%, while that by variable Y (PC2) was 24.04%.

**Figure 3:**
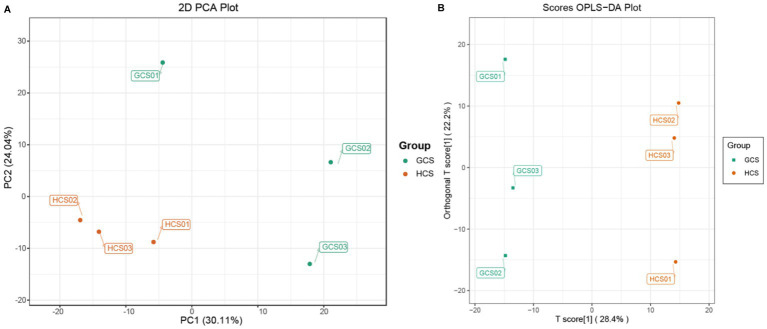
The information about the samples’ metabolome. The color of the points stands for the sample’s two groups. **(A)** PCA scores plot of the fleshy stems’ two ecotypes. PC1 stands for the first principal component, PC2 stands for the second principal component, and the interpretation rate of this principal component to the data set is expressed as a percentage. **(B)** OPLS-DA score plot of two ecotypes of the fleshy stems.

The PCA results showed that the samples of the two ecotypes had an obvious tendency to separate, indicating that the metabolite composition was different (Figure 3A). To furthermore analyze the discrepancies in metabolites between samples and seek out the distinct metabolites, we performed OPLS-DA, and the OPLS-DA score also showed noteworthy segregation between samples. In addition, the OPLS-DA scoring map was used to test the difference between the two parts (Figure 3B), and the model’s predicted value was greater than 50%. The OPLS-DA arrangement experiment also demonstrated this segregation. The OPLS-DA S-plot shows the significant difference of metabolites in samples (Supplementary Figures 2A–C).

Based on widely targeted metabolomics (WTM) techniques, we detected and identified 12 classes with add up to 876 metabolites, including 397 primary metabolites and 479 secondary metabolites. There were 164 lipids, 157 phenolic acids, 94 amino acids and derivatives, 87 flavonoids, 77 organic acids, 66 alkaloids, 62 nucleotides and derivatives, 37 lignans and coumarins, 33 terpenoids, 2 quinones, 1 steroid, and others (Supplementary Figure 3).

**Figure 4:**
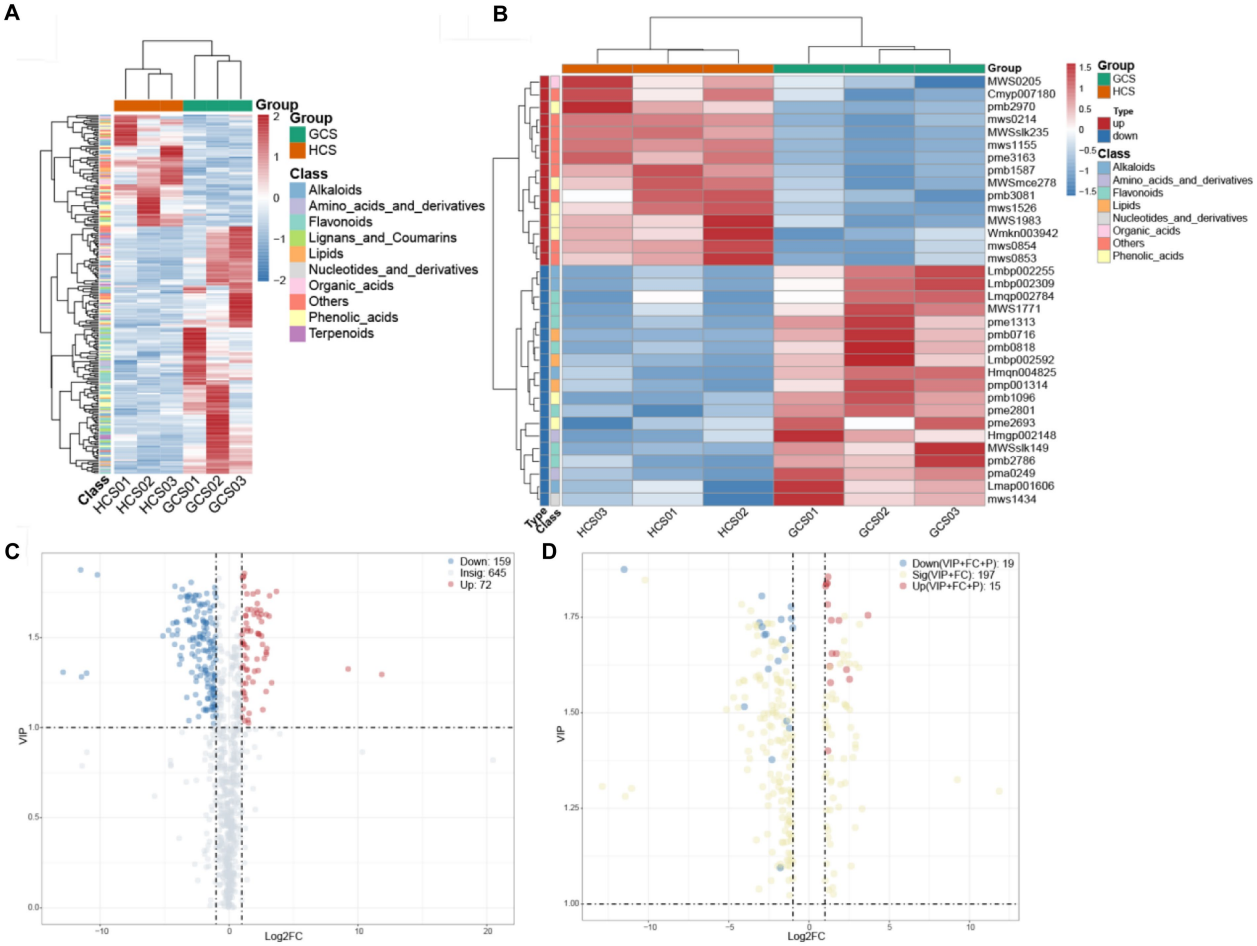
The changes in the differential metabolites. **(A,B)** The horizontal coordinate denotes diverse groups, the vertical coordinate denotes the metabolites, and the colored blocks at diverse locations denote the relative quantity of metabolites. **(A)** Heatmap of distinct metabolites. The metabolites for which VIP > 1 and FC ≥ 2, FC ≤ 0.5 were considered to be significantly changed. **(B)** Heatmap of key differential metabolites. Metabolite patterns were detected in two clusters. The metabolites for which VIP > 1, FC ≥ 2, FC ≤ 0.5, and *p* < 0.05 were deemed to be crux prominently variation. **(C,D)** Red represented the up-regulation of expression, blue represented the downregulation of expression, gray represented the insignificant of expression, and yellow represented the significance of expression. **(C)** The volcanic plot of differential metabolites. The metabolites for which VIP > 1 and FC ≥ 2, FC ≤ 0.5 were considered to be significantly changed. **(D)** The volcanic plot of key differential metabolites. The metabolites for which VIP > 1, FC ≥ 2, FC ≤ 0.5, and *p* < 0.05 were deemed to be crux prominently variation.

OPLS-DA can maximize the distinction between-group, which is beneficial to cast about for distinct metabolites. On account of the OPLS-DA’s results, the procured plenary analysis of the OPLS-DA model’s VIP values can preliminarily single out the metabolites with differences between groups. Meanwhile, the differential metabolites can be furthermore singled by associating the *p*-value or FC of single-variable analysis. The detected differential metabolites lead to positive or negative regulation of inter-group expression according to a certain threshold range. We screened 231 distinct metabolites between groups employing VIP ≥ 1 and FC ≥2 or FC ≤0.5 as the standards. There were 72 up-regulated and 159 down-regulated metabolites (Figures 4A,C).

Phenylethanoid glycosides (PhGs) are the classically medicinal ingredients in *Cistanche*. The PhGs metabolites that we detect in *Cistanche sinensis*, It mainly includes poliumoside, tubuloside A, verbascoside, isoacteoside, cistanoside A, echinacoside, and 2′-acetylacteoside. We found that echinacoside, isoacteoside, and cistanoside A were significantly downregulated at the sandy ecotype. There are no significant differences in sandy and loam ecotypes of poliumoside, tubuloside A, verbascoside, and 2′-acetylacteoside. In addition, poliumoside is a specific PhGs component in *Cistanche sinensis*.

On this basis, to further search for core differential metabolites, we screened 34 differential metabolites with a *p* <0.05 as the standard. There were 15 up-regulated and 19 down-regulated metabolites (Figures 4B,D).

Grouping metabolites with identical expression tendency into one group, hierarchical clustering shows two clusters (Figure 4B). In the upregulated cluster, numerously the 3/7 phenolic acids (4-hydroxy-3,5-diisopropyl benzaldehyde, sinapyl alcohol, and 3-hydroxy-4-isopropyl benzyl alcohol-3-*O*-glucoside), 2/7 flavonoids (nepetin-7-*O*-alloside and hesperetin-5,7-di-*O*-glucoside) and 1/1 organic acids (allantoin) were tested. The relative abundance of these metabolites at the sandy ecotype was lower than at the loam ecotype samples. In addition, 9 others (including 3-hydroxy-4-methoxy benzoic acid, 4-*O*-glucosyl-3,4-dihydroxy benzoyl alcohol, coniferin, syringin, isovitexin, isorhamnetin-3-*O*-Glucoside, 3’-*O*-methyltricetin-5-*O*-glucoside, kaempferol-3,7-di-*O*-glucoside, and tricin-4’-*O*-glucoside-7-*O*-glucoside) were less abundant at the sandy ecotype than at the loam ecotype samples. The down-regulated cluster primarily consisted of 4 phenolic acids (3-hydroxy-4-methoxy benzoic acid, 4-*O*-glucosyl-3,4-dihydroxy benzoyl alcohol, coniferin, and syringin), 5 flavonoids (isovitexin, isorhamnetin-3-*O*-glucoside, 3’-*O*-methyltricetin-5-*O*-glucoside, kaempferol-3,7-di-*O*-glucoside, and tricin-4’-*O*-glucoside-7-*O*-glucoside), 4 alkaloids (indole, N-acetyl putrescine, salicylamide, and methoxyindoleacetic acid), 3 lipids (9-hydroxy-10,12,15-octadecatrienoic acid, rabdosia acid A and 1-linolenoyl-rac-glycerol-diglucoside), 1 nucleotide and derivatives (7-methylxanthine) and 2 amino acids and derivatives (L-tyrosine methyl ester and N′-formyl kynurenine) (Supplementary Table 2). These ions were more abundant in the sandy ecotype than in the loam ecotype samples. *Cistanche sinensis* reflects differences in the metabolome of fleshy stem at two ecotypes, and furthermore, the concrete factors contributing to these differences require to identify.

### 3.3. Microbial community diversity, composition, and functional prediction of *Cistanche sinensis* fleshy stems was analyzed in Qingtongxia and Tongxin

Plant metabolites are considered candidates for shaping plant microorganisms, and influencing their components and function (Jacoby et al., 2020, 2021). Intriguingly, the plant microbiome also influences the metabolome of the host (Scherling et al., 2009; van de Mortel et al., 2012; Huang et al., 2014; Ryffel et al., 2016; Etalo et al., 2018). Research have shown that metabolites of the uniform officinal growing at diverse sites vary, which may be related to the diversity, composition, and function of microorganisms growing at diverse sites. In addition, natural *Bacillus* spec. Div. strains can boost plant growth and increase the yield of plant flavonoids (Koeberl et al., 2013; Huang et al., 2018). Therefore, in the cause of furthermore investigating whether the endophytic changes are accompanied by metabolite changes, we performed amplified sequencing on fleshy stem endophytes from *C. sinensis*.

**Figure 5:**
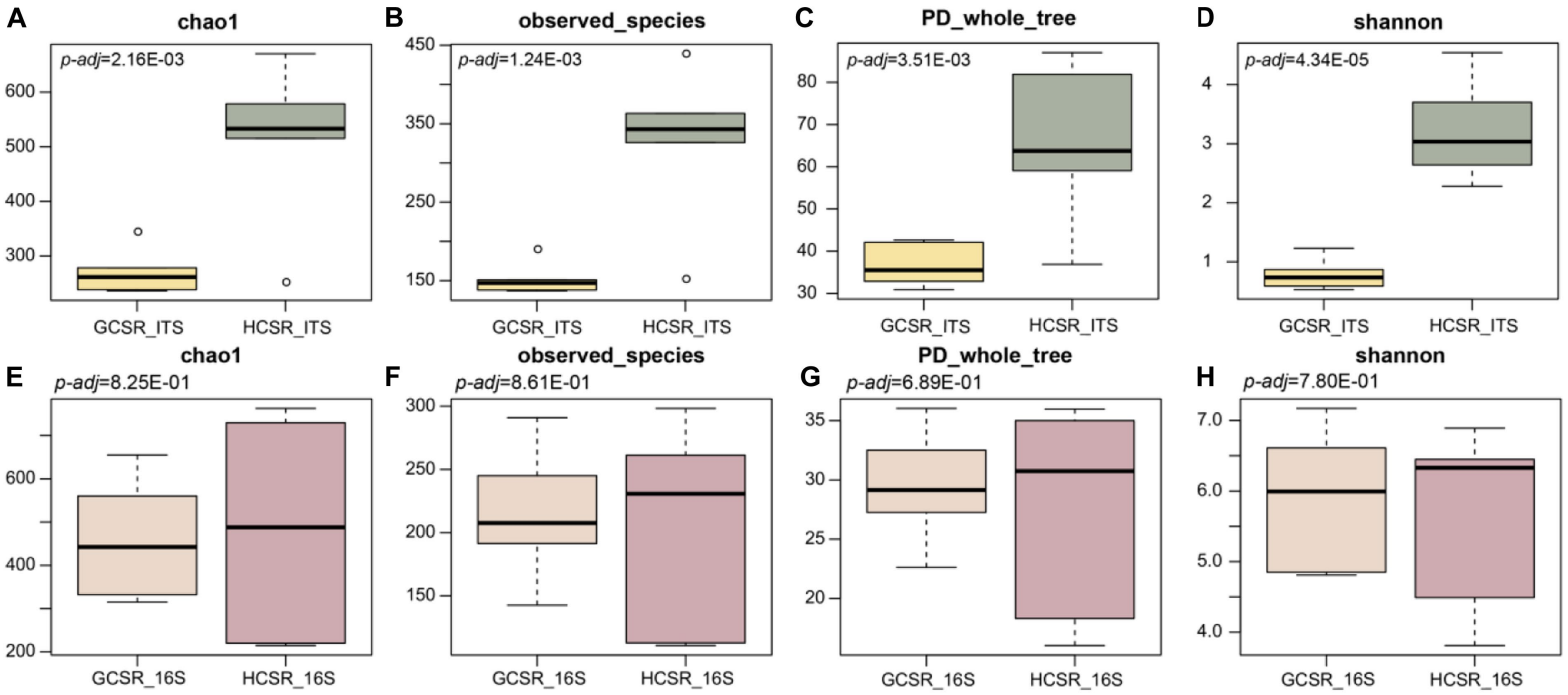
The microbiome communities’ *α* diversity in distinct sites (*p* < 0.05, ANOVA, Tukey-HSD test). **(A–D)** Results of fungi’s *α* diversity index. **(E–H)** Results of bacteria’s *α* diversity index. The box chart primarily involves 5 data nodes and arranges a set of data from largest to smallest to compute its upper edge, upper quartile, median, lower quartile, and lower edge, respectively.

We analyzed the bacteria and fungi’s diversity, community composition, and functional prediction at fleshy stems’ two ecotypes (sandy and loam ecotypes). We discovered *α* diversity parameters (Chao1, Observed species, PD whole tree, and Shannon indices) of the fungal microbiota of the sandy and loam ecotypes were significantly different (*p*<0.05) (Figures 5A–D). However, the *α* diversity parameters (Chao1, Observed species, PD whole tree, and Shannon indices) of the bacterial microbiota of the sandy and loam ecotypes were not significantly different (Figures 5E–H). *α* diversity index proved that the bacteria and fungi’s diversity levels at the loam ecotype were noticeably higher for those at the sandy ecotype, suggesting that *C. sinensis* fleshy stems attracted more bacteria and fungi at the sandy ecotype.

**Figure 6:**
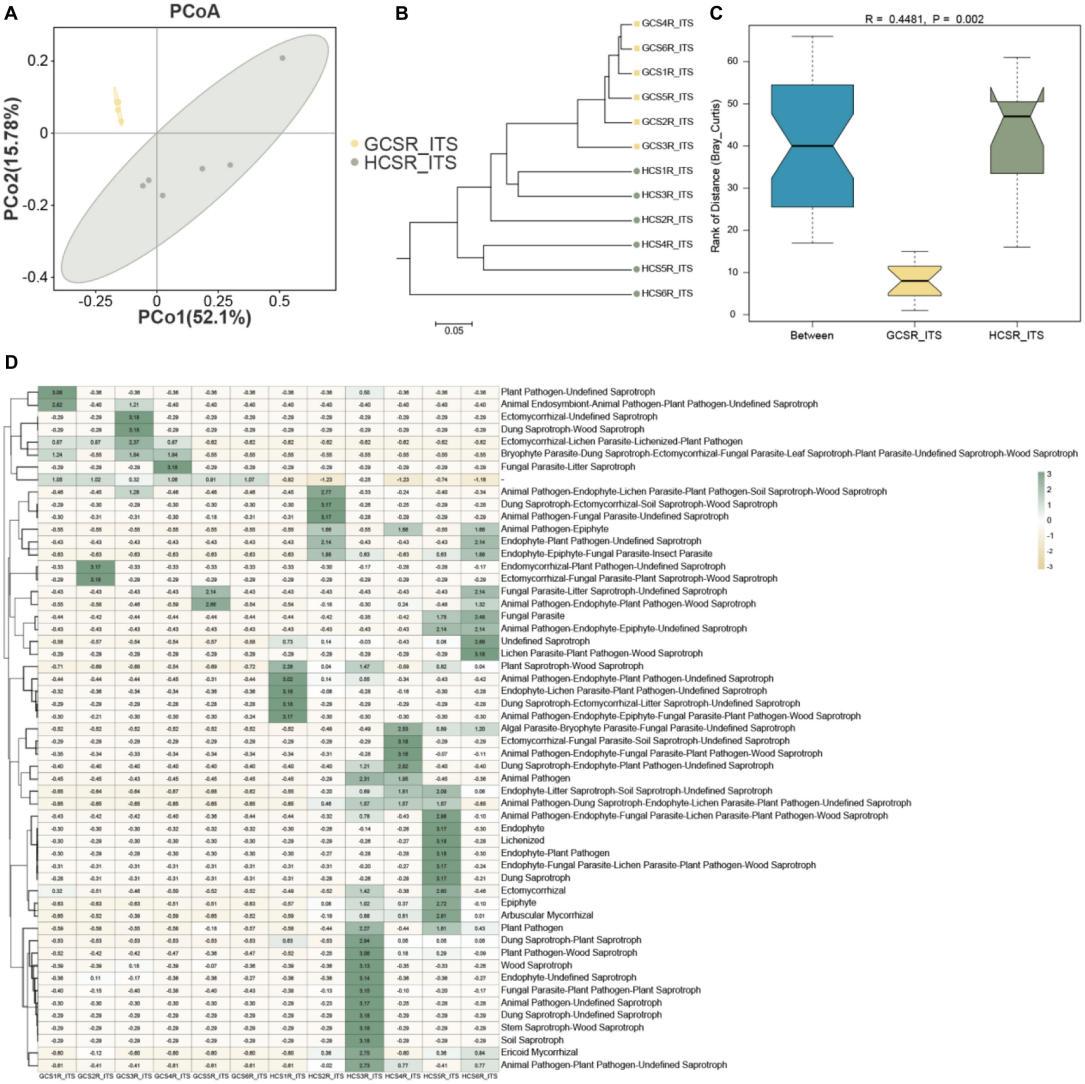
Endophytic fungal community diversity’s analysis in fleshy stems. **(A)** At the OTU level, the fungal community’s PCoA is on the strength of the bray Curtis. **(B)** UPGMA hierarchical clustering of fungi. Based on the bray Curtis, UPGMA hierarchical clustering was performed for two ecotypes. The closer the sample is, the shorter the length of the branches, showing that the two ecotypes’ species composition is more alike and can be brought together. **(C)** *β* distance data for fungi, based on the bray Curtis. **(D)** Heat map of fungal functional group (guilds).

**Figure 7:**
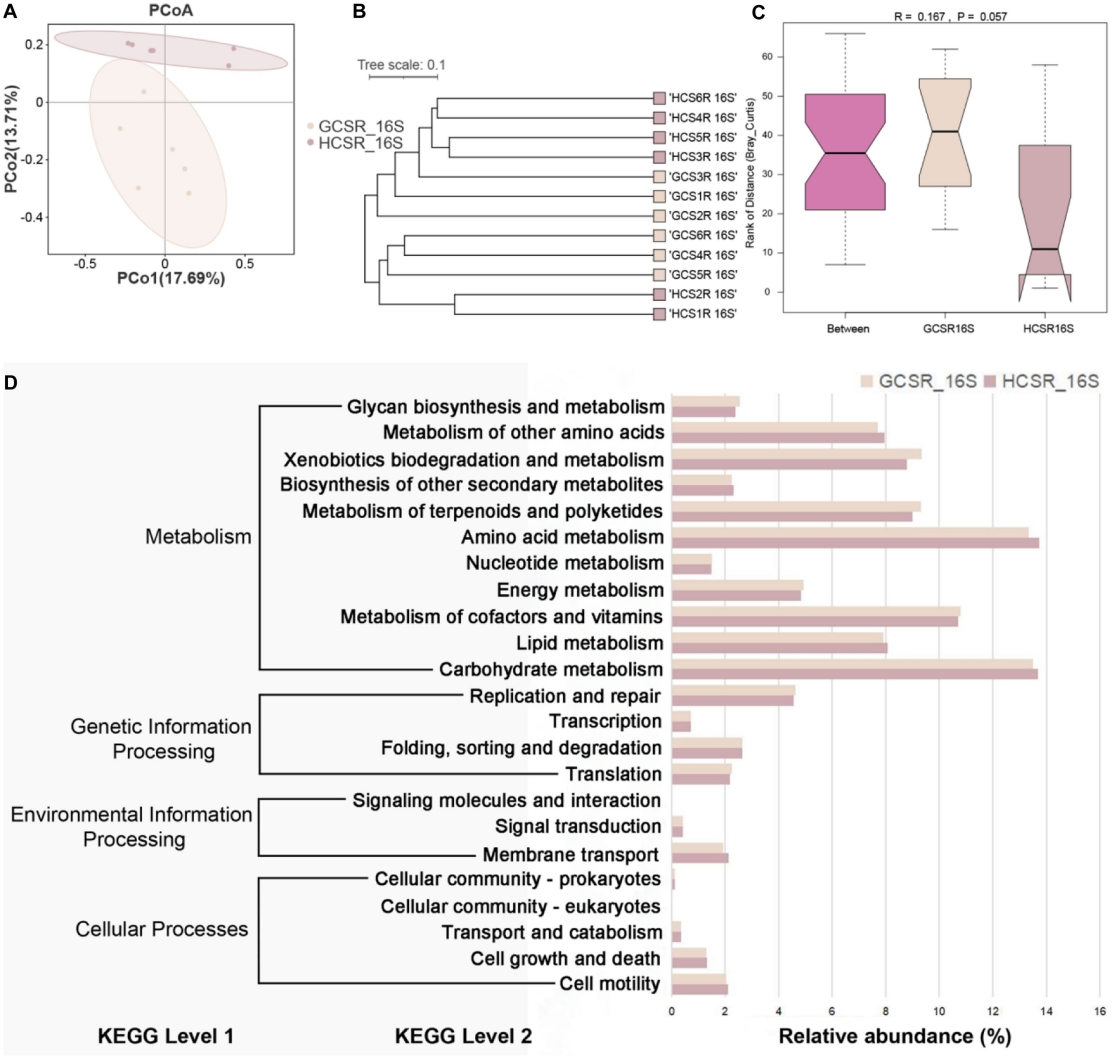
Endophytic bacterial community diversity’s analysis in fleshy stems. **(A)** At the OTU level, the bacterial community’s PCoA is on the strength of the bray Curtis. **(B)** UPGMA hierarchical clustering of bacteria. Based on the ray Curtis, UPGMA hierarchical clustering was performed for two ecotypes. The closer the sample is, the shorter the length of the branches, showing that the two ecotypes’ species composition is more alike and can be brought together. **(C)** *β* distance data for bacteria, grounded on the bray Curtis. **(D)** Extended bar graph predicting metabolic pathways in bacterial flora.

We also assessed the *β* diversity of fleshy stems’ endophytes at the sandy and loam ecotypes, contrasted the composition of the fleshy stems’ endophytes, computed bray-Curtis, and unifrac distances, and displayed the similarities or differences in sample community composition by PCoA (Figures 6A, 7A). On the side, we make use of the UPGMA algorithm to construct tree structures to describe and compare relationships between samples (Figures 6B, 7B). The bray-Curtis distance and unifrac distance’s PCoA of bacteria showed that sandy and loam samples took the shape of two obvious clusters, but the detachment was unsharpness (Figure7A). At the OTU level, PCo1 interpreted 17.69% of the overall aberrance, PCo2 interpreted 13.71%, and the accumulated aberrance interpreted by the two variables was 31.4%. The bray-Curtis and unifrac distances’ PCoA in fungi revealed distinct segregation in the samples (Figure 6A). At the OTU level, PCo1 interpreted 52.1% of the overall aberrance, PCo2 interpreted 15.7%, and the accumulated aberrance interpreted by the two variables was 67.8%. The samples’ hierarchical clustering was on the strength of the UPGMA, which overlapped on the PCoA plot. The PCoA displayed inapparent bacteria clustering (Figure 7B), while the fungi showed quite a strong clustering (Figure 6B). To be in favor of the fleshy stem fungi and bacteria’s clustering results acquired by PCoA, similarity analysis (ANOSIM) was implemented, and the consequences revealed that fungi at sandy and loam ecotypes were notably different (*R* = 0.448, *p* = 0.002) (Figure 6C), while the bacteria were not (*R* = 0.167, *p* = 0.057) (Figure 7C). Bacteria and fungi’s PCoA show that diverse growth ecotypes are the main provenience of changes in the composition of bacteria and fungi in *C. sinensis*.

**Figure 8:**
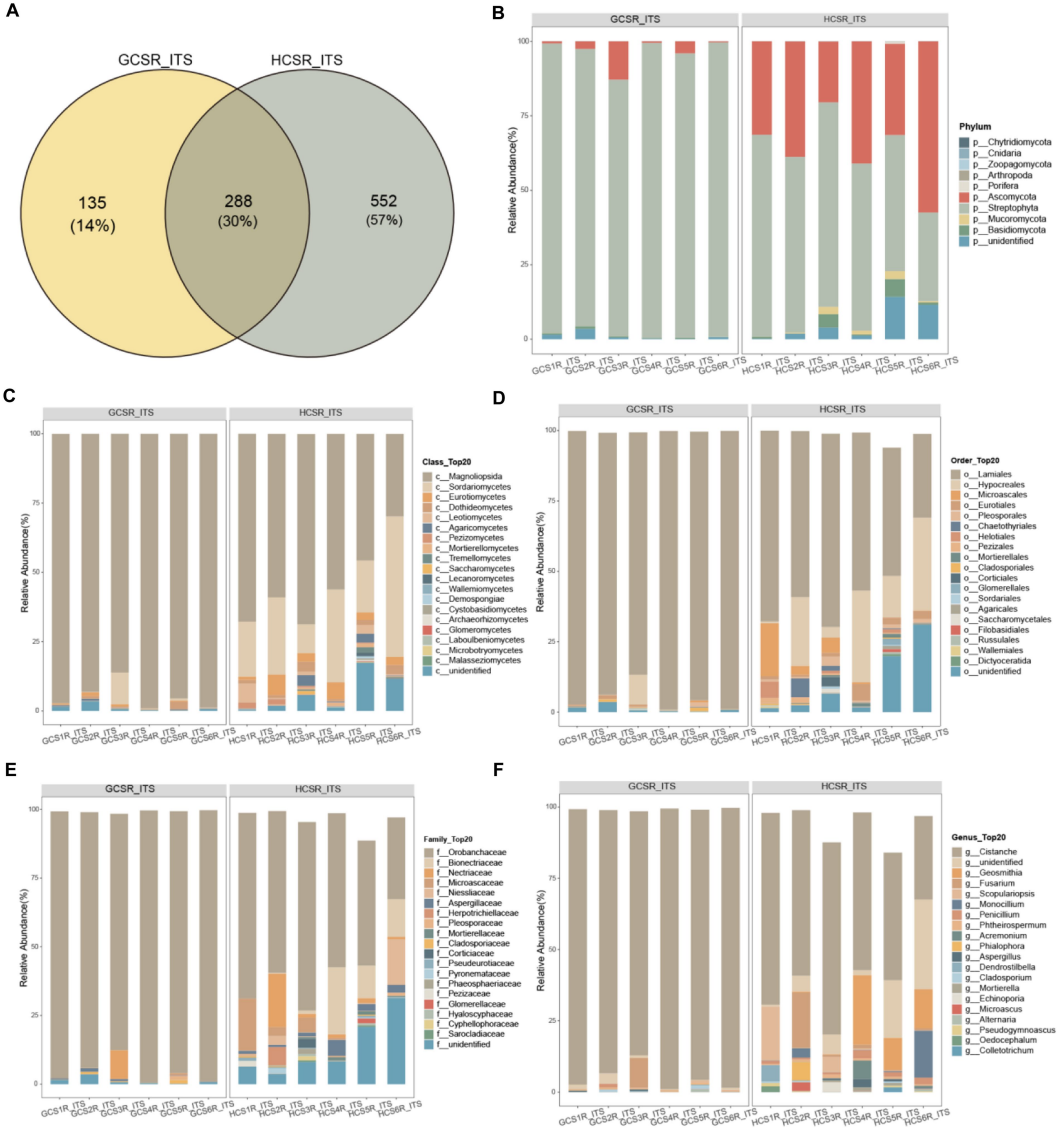
Taxonomic comparison of fungi at different taxonomic levels. The Venn diagram counted the number of fungal OTUs at two ecotypes, which could intuitively show the overlaps between groups and the number of fungal OTUs unique to each group. Selected fungal species with an abundance top 20 are shown in the figure and the void is the sum of the other fungal species. **(A)** Venn Diagram of overlap of fungal OTUs between groups. **(B)** At the phylum level the fungi’s relative abundance. **(C)** At the class level the fungi’s relative abundance. **(D)** At the order level the fungi’s relative abundance. **(E)** At the family level the fungi’s relative abundance. **(F)** At the genus level the fungi’s relative abundance.

**Figure 9:**
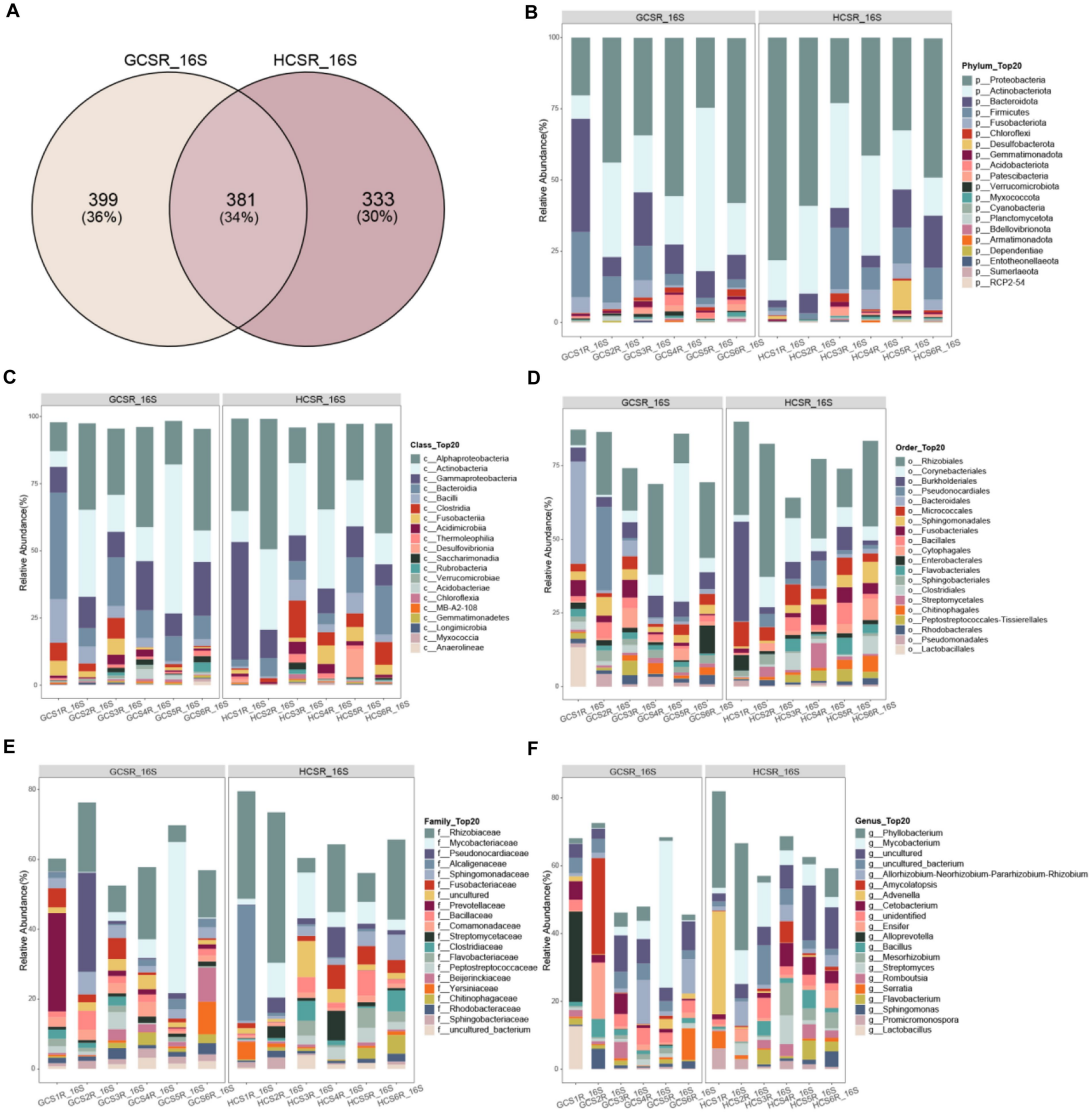
Distribution of bacterial species at different classification levels. The Venn diagram counted the number of bacterial OTUs at two ecotypes, which could intuitively show the overlaps between groups and the number of bacterial OTUs unique to each group. Selected bacterial species with an abundance top 20 are shown in the figure and the void is the sum of the other bacterial species. **(A)** Venn Diagram of overlap of bacterial OTUs between groups. **(B)** At the phylum level the bacteria’s relative abundance. **(C)** At the class level the bacteria’s relative abundance. **(D)** At the order level the bacteria’s relative abundance. **(E)** At the family level the bacteria’s relative abundance. **(F)** At the genus level the bacteria’s relative abundance.

For a preferable display of the endophyte diversity’s distribution in fleshy stems, we computed the ratio of OTUs in fleshy stems’ particular areas and the OTUs owned by diverse areas (Figures 8A, 9A). Add up to 14% of the OTUs for fungi were exclusive to the sandy and 57% were exclusive to loam. This indicates that the fungal community of loam is more abundant than sandy. However, there were few such differences in bacteria, for which add up to 36% of OTUs were specific to sandy and 30% to loam. We analyzed the comparison of fungi and bacteria species and relative abundance at diverse taxonomic levels.

At the phylum level of the fleshy stem endophytes’ relative abundance showed that the major bacteria were Proteobacteria, Actinobacteriota, Bacteroidota, and Firmicutes (Figure 9B; Supplementary Figure 4A), and the primary fleshy stem fungal microbiota was Streptophyta, Ascomycota, Basidiomycota and Mucoromycota (Figure 8B; Supplementary Figure 5A). Moreover, Verrucomicrobiota bacteria and Streptophyta fungi’s relative abundance were distinguished at sandy. Ascomycota and Mucoromycota fungi’s relative abundance was also remarkable diversity at loam (*p*<0.05) (Supplementary Tables 3, 4). For fungi at the class level, Sordariomycetes, Eurotiomycetes, Leotiomycetes, Dothideomycetes, Pezizomycetes, Mortierellomycetes, unidentified, Cystobasidiomycetes, and Tremellomycetes exhibited significantly a higher relative abundance at loam than at sandy. Magnoliopsida fungi’s relative abundance was notably upper in sandy than in loam, and Verrucomicrobiae bacteria were also significantly more abundant in sandy (Figures 8C, 9C; Supplementary Figures 4B, 5B).

At the order level, the top 10 fungi in relative abundance (Lamiales>Hypocreales>unidentified>Microascales>Eurotiales>Pleosporales>Chaetothyriales>Helotiales>Pezizales>Mortierellales) were significant difference between loam and sandy, and Bacteroidales bacteria differed significantly at two ecotypes (Figures 8D, 9D; Supplementary Figures 4C, 5C). Among the relative abundance of the top 20 fungi of the family level, there were significant differences in 12 dominant organisms. Among them, Orobanchaceae is significant at sandy ecotype, Bionectriaceae, Microascaceae, Niessliaceae, Aspergillaceae, Mortierellaceae, Bionectriaceae, Microascaceae, Niessliaceae, Corticiaceae, Pseudeurotiaceae, Pyronemataceae, Phaeosphaeriaceae, Glomerellaceae, Hyaloscyphaceae, and Cyphellophoraceae were significant at loam ecotype. And Prevotellaceae bacteria’s relative abundance was also a significant difference at the sandy ecotype (Figures 8E, 9E; Supplementary Figures 4D, 5D). In addition, the major bacteria at the genus level in the samples were *Phyllobacterium*, *Mycobacterium,* and *uncultured*, while the main fungal communities were *Cistanche*, *unidentified*, *Geosmithia,* and *Fusarium*. The relative abundance of *Phyllobacterium*, *uncultured,* and *Geosmithia* differed significantly at the loam ecotypes, as did the relative abundance of *Cistanche* fungi at sandy (*p*<0.05) (Figures 8F, 9F; Supplementary Figures 4E, 5E). These statistics showed that *C. sinensis* enlisted diverse endophytic bacteria and fungi from sandy and loam.

To know more about the effect of endophytes enlisted by *C. sinensis* at sandy and loam ecotypes, we forecasted the bacterial and fungal endophytes’ function.

FUNGuild was used to predict the trophic mode of the fungal community. Under conditions of highly probable and probable confidence ranking, the fungal community was classified into seven trophic mode groups, with pathotroph (0.5%), pathotroph-saprotroph (3.2%), pathotroph-saprotroph-symbiotroph (40.8%), pathotroph-symbiotroph (1.3%), saprotroph (6.5%), saprotroph-symbiotroph (5.2%) and symbiotroph (42.1%) being the trophic modes in the fungal community (Figure 6D). Besides, the fungal functional group’s composition was nonuniform. As a whole, we can observe that the functional abundance of fungi at the loam ecotype is higher. *Arbuscular Mycorrhizal* fungi were the maximum continually tested taxa with 114 OTUs (31.2%) member of Glomeraceae’s 9 known genera (such as *Glomus* and *Rhizophagus*). T-test was adopted to break down the diversities of the fungi-enriched guilds at two ecotypes, and the consequences indicated that there were 5 guilds with significant differences (*p* < 0.05), which were Endophyte-Litter Saprotroph-Soil Saprotroph-Undefined Saprotroph, Plant Saprotroph-Wood Saprotroph, Ectomycorrhizal-Lichen Parasite-Lichenized-Plant Pathogen, Endophyte-Epiphyte-Fungal Parasite-Insect Parasite, and Animal Pathogen-Dung Saprotroph-Endophyte-Lichen Parasite-Plant Pathogen-Undefined Saprotroph. Among them, only Ectomycorrhizal-Lichen Parasite-Lichenized-Plant Pathogen was more abundant at the sandy ecotype (Supplementary Table 5).

The PICRUSt2 analysis suggested the diversity of fleshy stem bacterial communities’ functional categories at two ecotypes. One hundred fifty-six KEGG orthologs (KOs) were noted by the KEGG database, displaying remarkable diversities between the two ecotypes (*p* ≤ 0.05). There are 4, 23, and 152 KEGG pathways annotated at the three levels (Level1, Level2, and Level3), respectively (Figure 7D). We calculated by the Kruskal and Wilcoxon tests that there was no remarkable diversity in the level1 and level2 pathways. At the level2, carbohydrate metabolism, amino acid metabolism, and metabolism of cofactors and vitamins were relatively highly abundant in the metabolism pathway; replication and repair were relatively highly abundance in the genetic information processing pathway; membrane transport was relatively highly abundance in the environmental information processing pathway; cell motility and cell growth and death were relatively high abundance in the cellular processes pathway. Meanwhile, there were significant differences in spliceosome (ko03040) and sulfur metabolism (ko00920) at the level 3 between the two ecotypes (*p* < 0.05), the relative abundance was higher at the sandy ecotype. The spliceosome is not a straightforward steady complex, but rather an actional family of molecules that package on the pre-mRNA and assist infold a construction that permits transesterylation to take place (Supplementary Table 6). These results suggest that the spliceosome may affect the biosynthesis of compounds in *C. sinensis*. Sulfur is an indispensable element to life and organic sulfur compounds’ metabolism plays a vital part in the worldwide sulfur circle. Sulfur oxidation capacity is fairly common in bacteria and archaea, including phototrophs and chemoautotrophs. This indicates that sulfur metabolism may affect energy metabolism during the growth and development of *C. sinensis*. Therefore, we speculate that the significant differences in spliceosome and sulfur metabolism of fleshy stems at sandy and loam ecotypes may be closely related to the habitat of *C. sinensis*.

### 3.4. Combined analysis of microbial and metabolite groups

Since some researchers have discovered that plant microbiome can straightway or mediately vary the synthesis of certain metabolites in viscumalbuni plants (Scherling et al., 2009; van de Mortel et al., 2012; Huang et al., 2014; Ryffel et al., 2016; Etalo et al., 2018). Microbiome analysis revealed that samples from sandy and loam attracted diverse endophytes, respectively, which may have different functions. Hence, one of the major causes for the signal diversity metabolomes in the samples may be the diversities in the microbiomes of the fleshy stem. We conducted microbe-metabolite spearman analysis and LEfSe to further explore this possibility.

**Figure 10:**
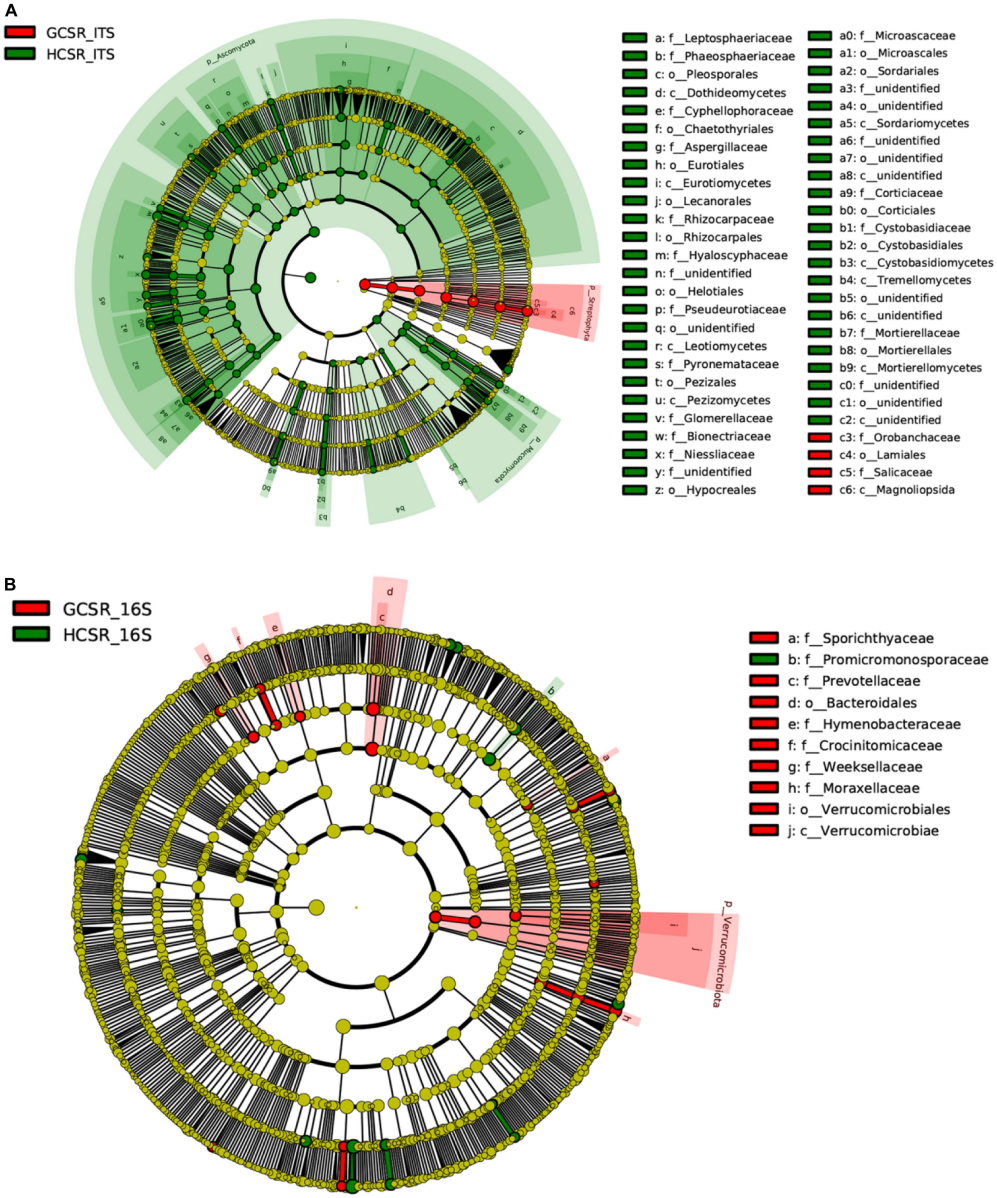
Evolutionary branching diagram of LEfSe analysis grounded on taxonomic information. The species with no noteworthy discrepancy were uniformly colored yellow, and the distinct species biomarkers were colored with the following groups. The red nodes represented the important microbial groups in the red group, and the green nodes represented the important microbial groups in the green group. **(A)** The fungal taxa’s cladogram. **(B)** The bacterial taxa’s cladogram.

LEfSe results revealed the bacterial and fungal biomarkers, which were the fleshy stem microbiota with statistical discrepancies between sandy and loam ecotypes. At the genus level, bacterial biomarkers at sandy were *Fluviicola*, *Chelativorans*, *Blastocatella*, *Rhodococcus*, *Anseongella,* and *Acinetobacter*, and bacterial biomarkers of the loam samples contained *Phyllobacterium*, *Promicromonospora*, *Arsenicitalea*, *Trichococcus* and *Acidovorax* (Figure 10B and Supplementary Figure 6). The sandy sample’s fungal biomarkers had *Cistanche*, and fungal biomarkers at loam were *Geosmithia*, *Scopulariopsis*, *Monocillium*, *Rhizocarpon*, *Pyrenochaeta*, *Penicillium*, *Acremonium*, *Echinoporia*, *Filobasidium*, *Mortierella*, *Microascus*, *Cystobasidium*, *Cyphellophora*, *Leptosphaeria*, *Clonostachys*, *Pseudogymnoascus*, *Phaeosphaeria*, *Trichosporon*, and *Colletotrichum* (Figure 10A; Supplementary Figure 7). The multiplicity and significant abundance of the above species may be the key factors that lead to the differences in community structure among taxa. The aforementioned consequences showed that the fungal diversity of loam was significantly higher than sandy. The bacterial diversity of sand was slightly lower than loam. This was consistent with the results of fungal and bacterial diversity at the two ecotypes in the previous analysis.

**Figure 11:**
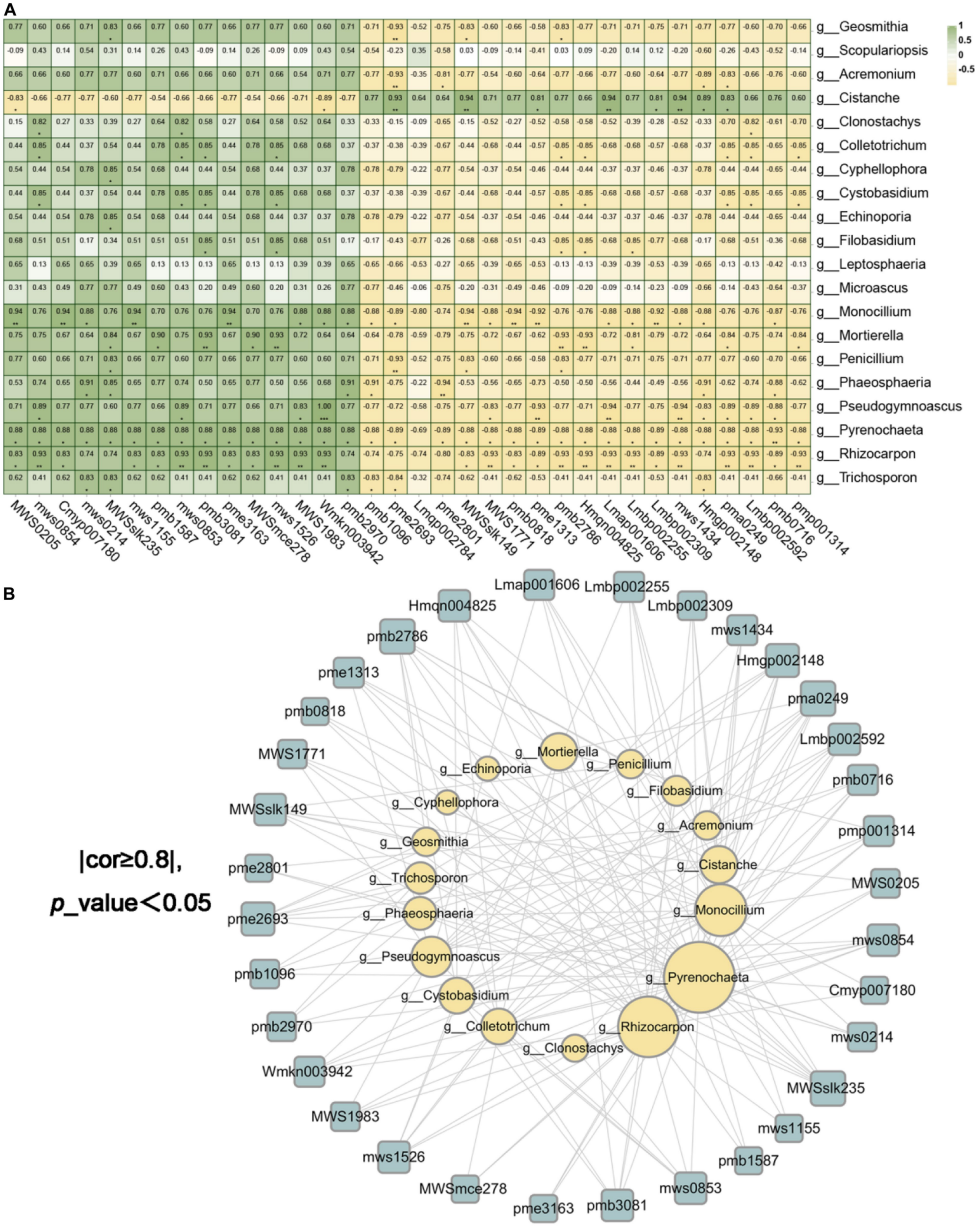
The endophytic fungi were associated with differential metabolites. **(A)** was the correlation heatmap based on Spearman analysis, revealing the correlation between 20 different endophytic fungi and 34 different metabolites at the genus level. The color scale indicates the correlation coefficient between endophytic fungi and metabolites. “*” on behalf of “*p* <0.05,” “**” on behalf of “*p* <0.01” and “***” on behalf of “*p* <0.001.” **(B)** were the endophytic fungi and metabolite network screened based on **(A)** (cor ≥ 0.8 and *p* <0.05).

**Figure 12:**
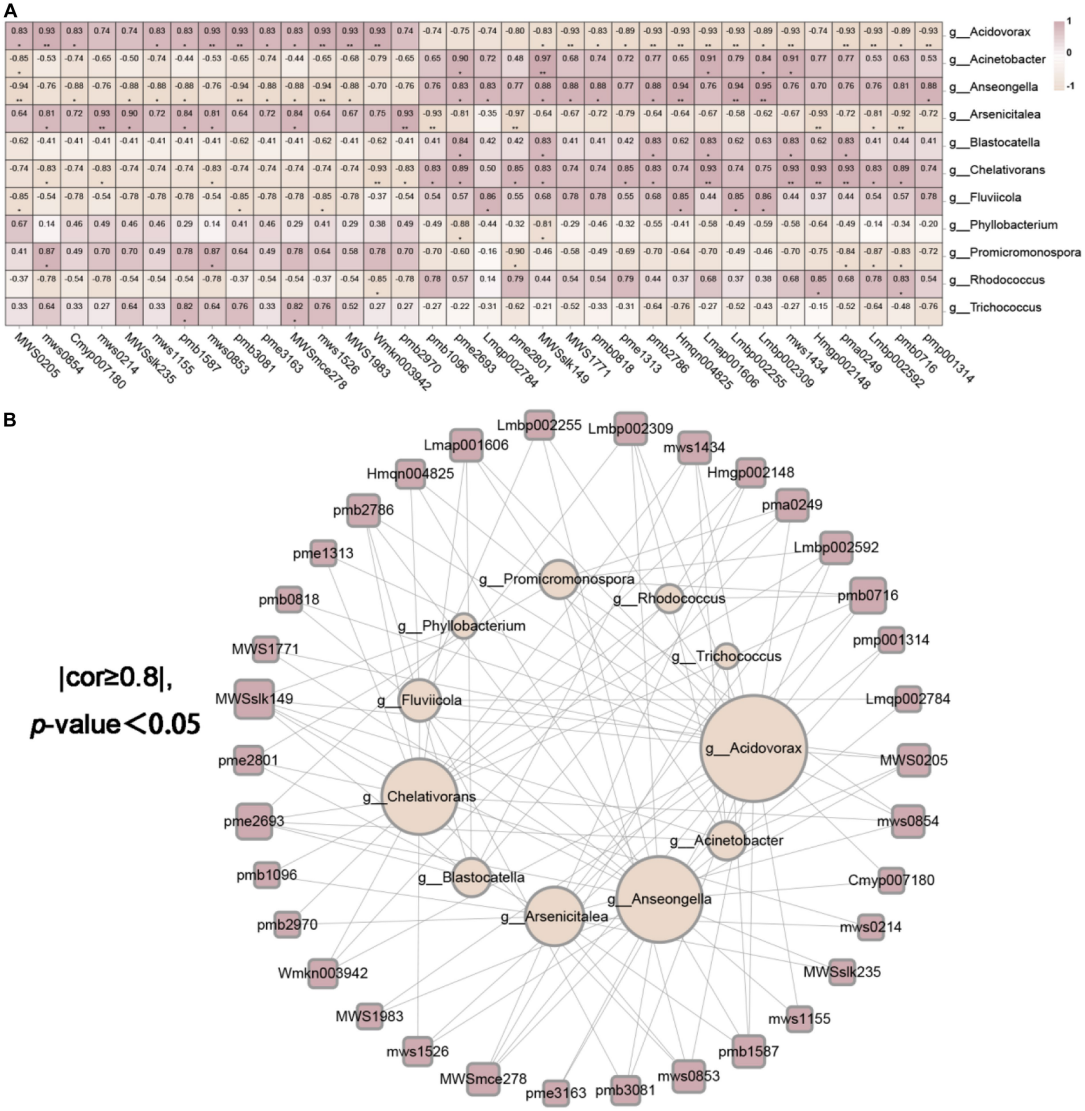
The endophytic bacteria were associated with differential metabolites. **(A)** was the correlation heatmap based on Spearman analysis, revealing the correlation between 11 different endophytic bacteria and 34 different metabolites at the genus level. The color scale indicates the correlation coefficient between endophytic bacteria and metabolites. “*” on behalf of “*p* <0.05,” “**” on behalf of “*p* <0.01” and “***” on behalf of “*p* <0.001.” **(B)** was the endophytic bacteria and metabolite network screened based on **(A)** (cor ≥ 0.8 and *p* <0.05).

We had access to Spearman correlation analysis to investigate the bacterial and fungal biomarkers at the genus level and the identified differential metabolites (Figures 11A,B, 12A,B). As shown in Figures 11A,B, which makes clear that the endophytic fungi notably relevance to the diversities in metabolites (*p* < 0.05 and correlation >0.8) were mainly *Pyrenochaeta*, *Rhizocarpon*, *Monocillium*, *Pseudogymnoascus* of Ascomycota, *Mortierella* of Mucoromycota and *Cistanche* of Streptophyta (degree ≥10, Supplementary Tables 7, 8). The endophytic bacteria notably relevant to the diversities in metabolites (*p* < 0.05 and correlation >0.8) were mainly *Acidovorax*, *Arsenicitalea*, *Chelativorans* of Proteobacteria, *Anseongella* of Bacteroidota (degree ≥10, Supplementary Tables 9, 10). Further, we screened that the correlation between the bacterial and fungal biomarkers and differential metabolites was greater than 0.95, and *Anseongella* was positively correlated with syringin (Lmbp002309). *Arsenicitalea* is negatively correlated with 7-methylxanthine (pme2801) and *Pseudogymnoascus* is completely positively correlated with nepetin-7-*O*-alloside (Wmkn003942) (cor =1). Accordingly, there is a familiar connection between endophytes and plant metabolites, which may be due to the metabolites’ production by endophytes, the effect on the production of host secondary chemicals, or more complicated mutual effects between the host and microorganisms (Hoyt et al., 2015; Singh et al., 2021; He et al., 2022).

## 4. Discussion

The growing development of plants is bound up with the plant microbiome internal to the plant (Turner et al., 2013; Vandenkoornhuyse et al., 2015; Sasse et al., 2018). Intriguingly, plant metabolites can mold the plant microbiome, and the plant microbiome can also influence the metabolome of the hosts (Scherling et al., 2009; van de Mortel et al., 2012; Huang et al., 2014; Ryffel et al., 2016; Etalo et al., 2018). The correlative research between metabolome and microbiome will define an in-depth comprehension of the important connections between particular metabolites and microbiome (Lee and Mazmanian, 2010; Lebeis et al., 2015; Beckers et al., 2017).

*Cistanche sinensis* is a noted Chinese Psammophytes with great medicinal value in northwest China. *Cistanche sinensis* fleshy stems are the mainly crude ingredients of certain Chinese medicines, and *C. sinensis* had the effect of windbreak and sand fixation. Previous studies on *C. sinensis* have focused on the molecular and compound identification of *Cistanche* (Tu et al., 2007; Shi et al., 2009; Gao et al., 2019; Liu et al., 2019, 2020; Song et al., 2019; Miao et al., 2022). This study adds to our understanding of the relationship between *Cistanche sinensis*’s metabolites and endophytes.

### 4.1. Metabolomic changes in the samples

In our study, the metabolites detected and identified in *C. sinensis* mainly focus on lipids, phenolic acids, and amino acids, especially phenolic acids in the secondary metabolites. The metabolome analysis showed that most metabolites’ relative abundance in sand was significantly higher than in loam, and 34 extremely significant differential metabolites were tested. The pivotal constituent, including echinacoside, isoacteoside, and cistanoside A were significantly downregulated in the sandy samples. Poliumoside is the unique metabolite of *C. sinensis* has no significant difference between the two ecotypes, which may be due to the close distance between the two ecotypes. There is limited research on whether different regions lead to differences in the medicinal ingredients of *C. sinensis*.

Surprisingly, we found that oleic acid, quercetin-7-*O*-glucoside, tricin-4’-*O*-glucoside, 3-*O*-feruloyl quinic acid, tricin-4’-*O*-glucoside-7-*O*-glucoside, dihydrochalcone-4’-*O*-glucoside, trifolirhizin (maackiain-3-*O*-glucoside) were detected only at the sandy samples, oleic acid, quercetin-7-*O*-glucoside, tricin-4’-*O*-glucoside, 3-*O*-feruloyl quinic acid, tricin-4’-*O*-glucoside-7-*O*-glucoside were notably down-regulated in the sandy samples. Hydroxy-3-methyl pentane-1,5-dioic acid, formononetin (7-hydroxy-4′-methoxy isoflavone), 2-aminophenol, lysoPC 20:5 were detected only in the loam samples, 2-aminophenol, lysoPC 20:5 were significantly up-regulated in the sandy samples. We speculated that tricin-4’-*O*-glucoside-7-*O*-glucoside can be used as a signature component to distinguish sandy and loam ecotypes.

Combined with the previous research basis of the team, HPLC characteristic maps of *Cistanche* were changed with great differences in similarity due to different primitive (Wang et al., 2019). The similarity between the characteristic maps of *C. sinensis* and *Cistanche deserticola* is less than 0.300, the difference is obvious, and the two can be completely distinguished by similarity (Wang J. et al., 2021). In addition, previous studies have shown that geniposide, poliumoside and brandioside are specific components of *C. sinensis*. In this study, no geniposide and geniposide were found in the metabolites of *C. sinensis*, which may be because the chemical components of Chinese herbal medicine are greatly affected by growth years, growing environment, and harvesting time.

### 4.2. Microbiota changes in the samples

We studied endophytes at the sandy and loam ecotypes, the consequences showed that *α* and *β* diversity of the endophytic bacteria sandy and loam ecotypes was not notable discrepancy. By comparison, the endophytic fungi’s *α* and *β* diversity was notably a discrepancy.

Phylum, class, order, family, and genus-level distribution of fleshy stem endophytes from sandy and loam indicated that the endophytes’ species compositions were the identity, whereas the species diversity of loam samples was higher than sandy samples in endophytic fungi; there was no notable discrepancy in species diversity between loam and sandy samples, and sandy samples were slightly higher than loam samples in endophytic bacteria.

Functional prediction using PRICRUSt2 and FUNGuild showed that symbiotroph (42.1%) was the main trophic mode in the fungal community. We can observe that the functional abundance of fungi at the loam ecotype is higher. Undefined Saprotroph and Arbuscular Mycorrhizal are annotated to the more abundant fungi guild. Undefined saprotroph may increase the risk of plant diseases and is a pathogenic factor (Li et al., 2021). The functional prediction indicated that the endophytes’ functional compositions were miscellaneous from the two ecotypes. *Cistanche sinensis* absorbed different fleshy stem endophytes in the two circumstances, and differences in fleshy stem endophytes may have led to differences in the fleshy stem endophytes’ function. Arbuscular mycorrhizal helps plants grow and protect them from all kinds of environmental stresses (Lenoir et al., 2016). *Arbuscular mycorrhizal* fungi colonize plant root systems and regulate plant growth in a variety of ways (Evelin et al., 2009). The spliceosome and sulfur metabolic pathway is the key functions of endophytic bacteria in the samples of two ecotypes and may be closely related to the growing environment of *C. sinensis*. The discrepancy in fleshy stem endophytes may be associated with the disparate growth circumstances of the two ecotypes. According to the field investigation results of our team, the closeness of human activities may be an essential factor affecting the microbial diversity of *C. sinensis*. The loam ecotype is relatively close to the human range, while the sandy ecotype is the Pigeon Mountain site, which is largely uninhabited. Therefore, there are many reasons why *C. sinensis* can recruit endophytes with different functions. This research mainly studies the relationship between endophytes and metabolites.

### 4.3. Correlation of microbial and metabolite

At the genus level, LEfSe consequences revealed GCS samples involved biomarkers of 6 bacteria and 1 fungi, while the HCS samples involved biomarkers of 6 bacteria and 23 fungi. The microorganisms represented by these biomarkers may be a strategy for the survival of *C. sinensis* in distinct circumstances. The research and application of microorganisms in distinct surrounding conditions can refer to the results of LEfSe.

The relationship between microbes and metabolites makes it difficult to explain whether metabolites influence the microbial composition, or whether these microbes alter metabolite synthesis. The fleshy stem microflora is associated with the linked differential metabolites, with noteworthy discrepancies between the two ecotypes, and it is more likely that the underlying fleshy stem microflora causes the metabolomic discrepancies. Therefore, by uniting the consequences of Spearman and LEfSe, we selected the underlying microbiota of 11 discrepant bacteria and 20 discrepant fungi, which presumably affected the metabolome in *C. sinensis* at the genus level. Surprisingly, the results of the high correlation between microbes and metabolites are consistent with the hypothesis that microbes influence the synthesis of compounds, and vice versa.

In short, our research can offer a tactic for the study and adhibition of *C. sinensis* and other officinal plants. Nevertheless, the microorganisms’ effect in Chinese herbs on host metabolites snot been in depth studied (Huang et al., 2018), and our research may provide strategies to relieve stress. Such as, to improve the quality of *C. sinensis* to raise the relative amount of some alkaloids, adding or reducing the level of some microorganisms based on Spearman results can be taken into account. Similar studies in other plants could use these microbes as subjects. In addition, other Chinese herbal medicine studies can also learn from our methods to explore important microorganisms associated with medicinal ingredients. Admittedly, our results are based on bioinformatics analysis only. Thus, not all potential fleshy stem microbiota that we have indicated can change metabolite synthesis in *C. sinensis*. In the future, further experiments are needed to isolate and identify these potential fleshy stem microbiota and explore related functions.

## 5. Conclusion

Free radicals in the human body can seriously damage human cells and tissues, and then cause chronic diseases and aging effects, appropriate supplement antioxidant drugs and food to eliminate free radicals in the human body, enhance the antioxidant defense system, delay aging, and prevent disease health methods. *In vitro*, antioxidant experiments showed that *C. sinensis* with loam ecotype had a better antioxidant effect. Furthermore, combining the metabolome with the microbiome can assist us in sinking our comprehension of the relationship between plants and their microbiome, to find microorganisms that can relate to their host’s metabolites. *Cistanche sinensis* fleshy stem attracted different bacteria and fungi, and both bacteria and fungi presumably implemented different functions in the host’s fleshy stems. Key correlation results that *Anseongella* was positively correlated with syringin, *Anseongella*, a bacterium associated with refractory macromolecules, promotes xenobiodegradation and metabolic pathways, as well as biosynthesis of other secondary metabolites, which are closely related to lignocellulosic degradation and humus formation, and can improve soil fertility. Syringin is a biologically active chemical compound with anti-inflammatory, oxidation resistance, and neuroprotection. *Arsenicitalea* is a novel arsenic-resistant bacterium, which has a certain tolerance to heavy metals and is beneficial to soil remediation. Compared to other xanthines in clinical use, such as caffeine and theobromine, 7-methylxanthine is the first molecule found to be effective in treating myopia in phase II clinical trials. *Arsenicitalea* is negatively correlated with 7-methylxanthine. The capacity of a pathogen to last outer the host, to live as an “environmental reservoir,” can aggravate the effect of the illness and enhance the possibility of host disappearance. *Pseudogymnoascus destructans*, the fungal pathogen, which leads to white-nose syndrome in bats, has been discovered in cavern soil in time of the summer months when bats hibernate. The *Pseudogymnoascus pathogenesis* appears to be a complex adaptation of fungus in its abiotic (caves and mines) and biotic (bats) environments. *Pseudogymnoascus* is completely positively correlated with nepetin-7-*O*-alloside. Therefore, there may be a causal relationship between *Pseudogymnoascus* and nepetin-7-*O*-alloside, when the relative abundance of *Pseudogymnoascus* increases, the content of nepetin-7-*O*-alloside will add correspondingly. *Cistanche sinensis* and some Chinese herbs confront several issues, incorporating lack of research into how metabolites interact with the plant microbiome (Huang et al., 2018), the wild resources have decreased sharply, and the factitious cultivation’s experience and introduction is not enough. These issues have contributed to stress on cultivation and relevant drug research. We managed our research to offer academic support for the effective cultivation of *C. sinensis* in the prospect.

## Data availability statement

The datasets presented in this study can be found in online repositories. The names of the repository/repositories and accession number(s) can be found at: NCBI-PRJNA933952.

## Author contributions

LH, ML, and MZ planned and designed this research and revised the manuscript. YM and XZ collected the samples. MZ carried out experiments and wrote the manuscript. MZ, XS, and YM performed the analysis and visualization of the data. All authors contributed to the article and approved the submitted version.

## Funding

This work was financially supported by the National Natural Science Foundation of China (82073960, 82274045, and U1812403-1), National Science and Technology Fundamental Resources Investigation Program of China (2018FY100701), the Open Fund of State Key Laboratory of Southwestern Chinese Medicine Resources (SKLTCM2022015) and CAMS Innovation Fund for Medical Sciences (CIFMS, 2022-I2M-1-017). The authors also acknowledge assistance from medical writers, proof-readers, and editors.

## Conflict of interest

The authors declare that the research was conducted in the absence of any commercial or financial relationships that could be construed as a potential conflict of interest.

## Publisher’s note

All claims expressed in this article are solely those of the authors and do not necessarily represent those of their affiliated organizations, or those of the publisher, the editors and the reviewers. Any product that may be evaluated in this article, or claim that may be made by its manufacturer, is not guaranteed or endorsed by the publisher.

## Abbreviations

UPLC-MS/MS, Ultra-high-performance liquid chromatography-high-resolution mass spectrometry
PhGs, phenylethanoid glycosides
NT, near threatened
QC, quality control
IS, ion spray
CUR, curtain gas
OTUs, operational taxonomic units
PCA, principal component analysis
HCA, hierarchical cluster analysis
FC, fold change
OPLS-DA, orthogonal partial least squares-discriminant analysis
WTM, widely targeted metabolomics
VIP, variable importance in the projection
KOs, KEGG orthologs
UPGMA, unweighted pair group method with arithmetic mean
LDA, Effect Size (LEfSe)
