# Supplementary material for: Revealing ecotype influences on Cistanche sinensis: from the perspective of endophytes to metabolites characteristics
Source: Front Microbiol. 2023 Jun 27;14:1154688. doi: 10.3389/fmicb.2023.1154688 (PMC10394521; doi:10.3389/fmicb.2023.1154688)
Supplement: Supplementary file 1 [file Data_Sheet_1.zip › Supplementary figures.DOCX]

Supplementary Material

Revealing Ecotype Influences on *Cistanche Sinensis*: From the Perspective of Endophytes to Metabolites Characteristics

**Min Zhang^1,2^, Yujing Miao^2^, Xinke Zhang^2^, Xiao Sun^2^, Minhui Li^1,3,4*^ and Linfang Huang^2*^**

**Correspondence:** Minhui Li: prof_liminhui@yeah.net; Linfang Huang: lfuang@implad.ac.cn

# Supplementary Figures


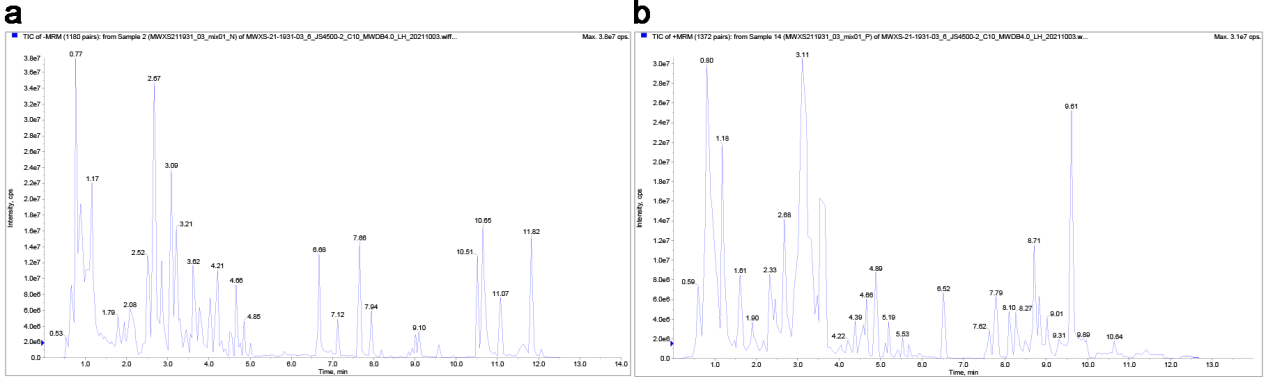


**Supplementary Figure 1.** Total ion chromatogram (TIC) analysis by mixed quality spectrum. (a) Negative ion mode. (b) Positive ion mode.


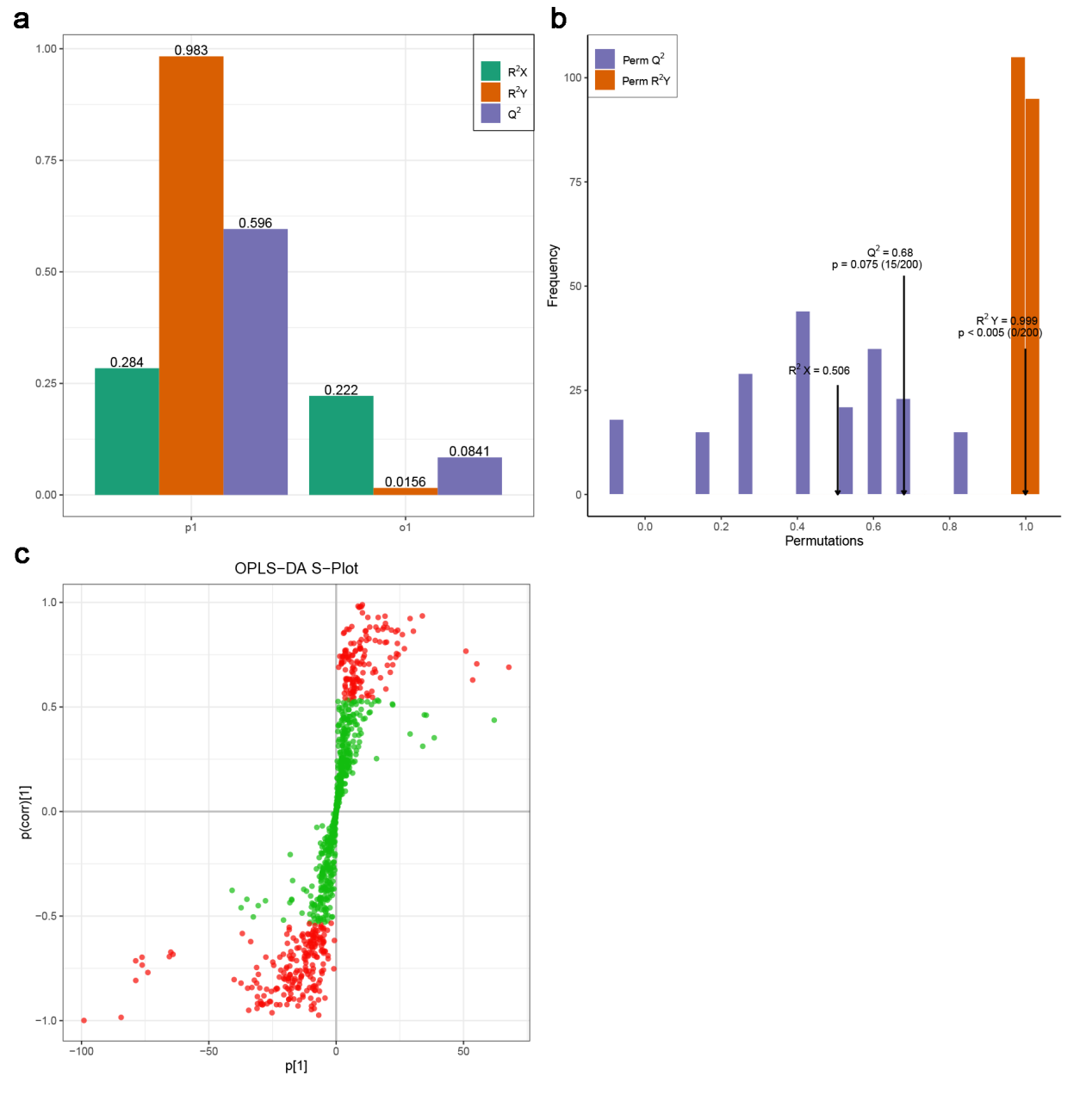


**Supplementary Figure 2.** (a) OPLS-DA model overview. (b) The OPLS-DA model was verified by the permutation test. (c) OPLS-DA S-plot.


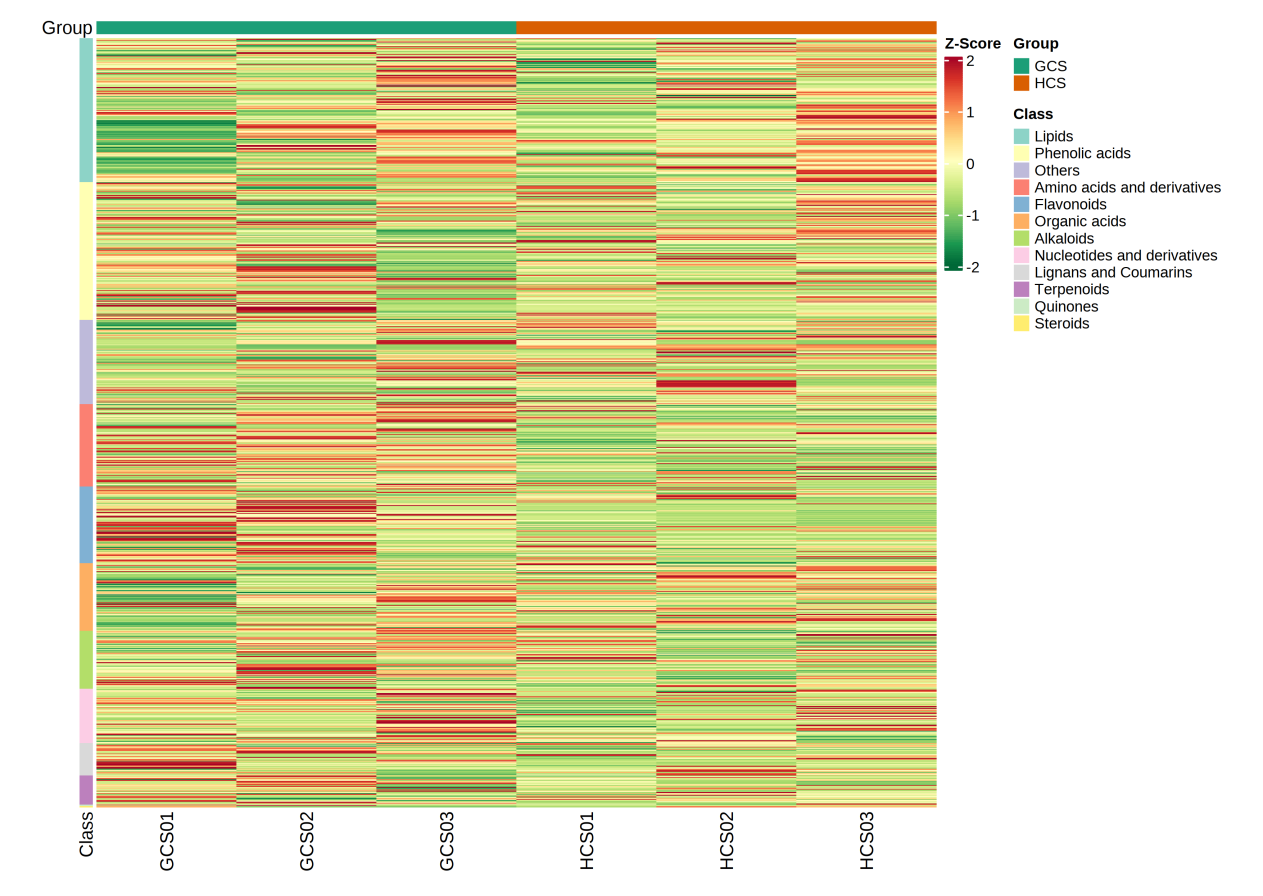


**Supplementary Figure 3.** The cluster heatmap of all metabolites. The color scale indicated the abundance of metabolites. Different colors are the values obtained after standardized treatment of relative content (red represented high content, green represented low content). Horizontal is sample name, vertical is metabolite information.


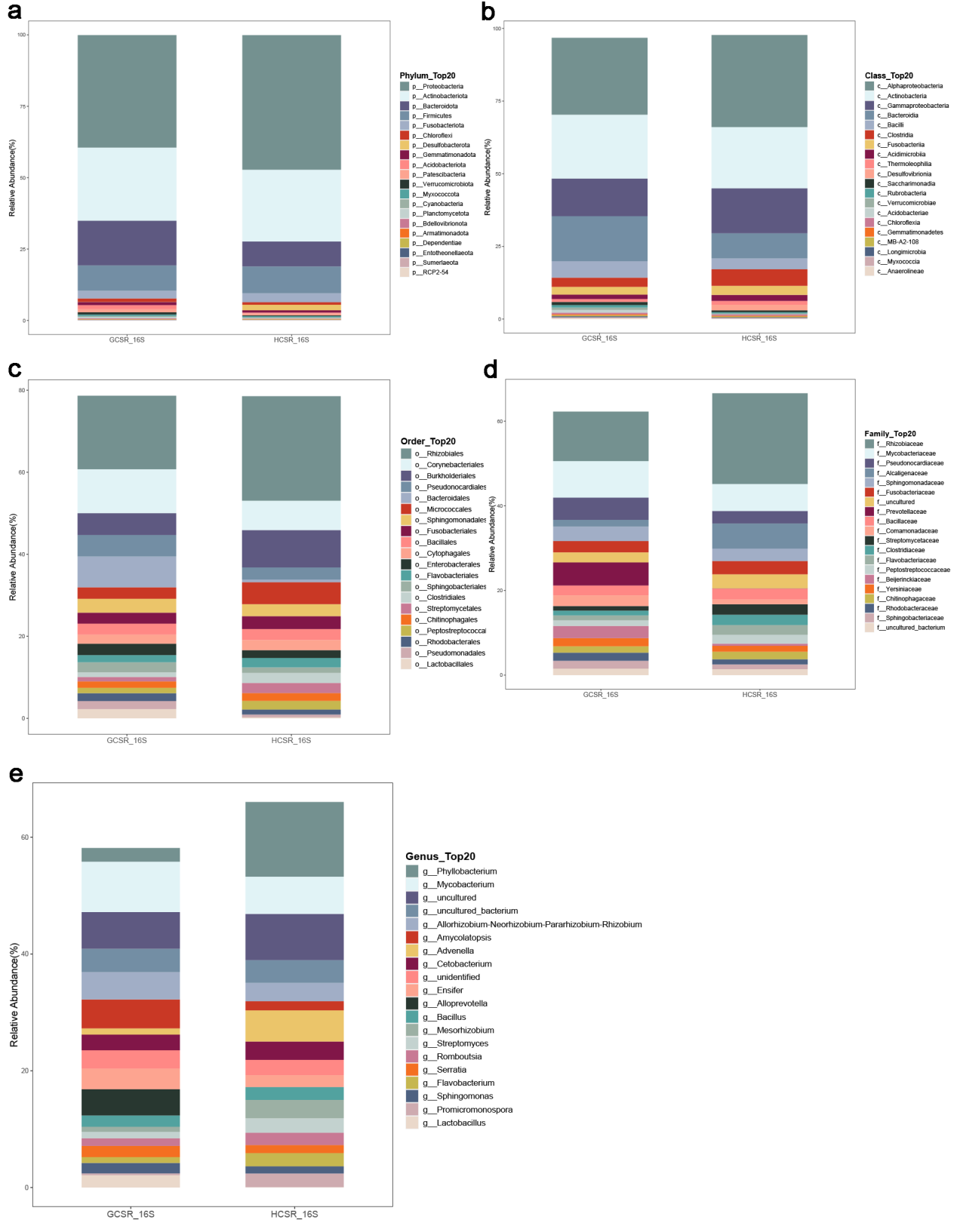


**Supplementary Figure 4.** Distribution of bacterial species at different classification levels by groups. Selected bacterial species with an abundance top 20 are displayed in the figure and combined other bacterial species into vacancy; “unidentified” represents the bacterial species without taxonomic annotation. Different bacterial communities are distinguished by different color combinations. (a) Relative abundance of bacteria at the phylum level. (b) Relative abundance bacteria at the class level. (c) Relative abundance bacteria at the order level. (d) Relative abundance bacteria at the family level. (e) Relative abundance bacteria at the genus level.


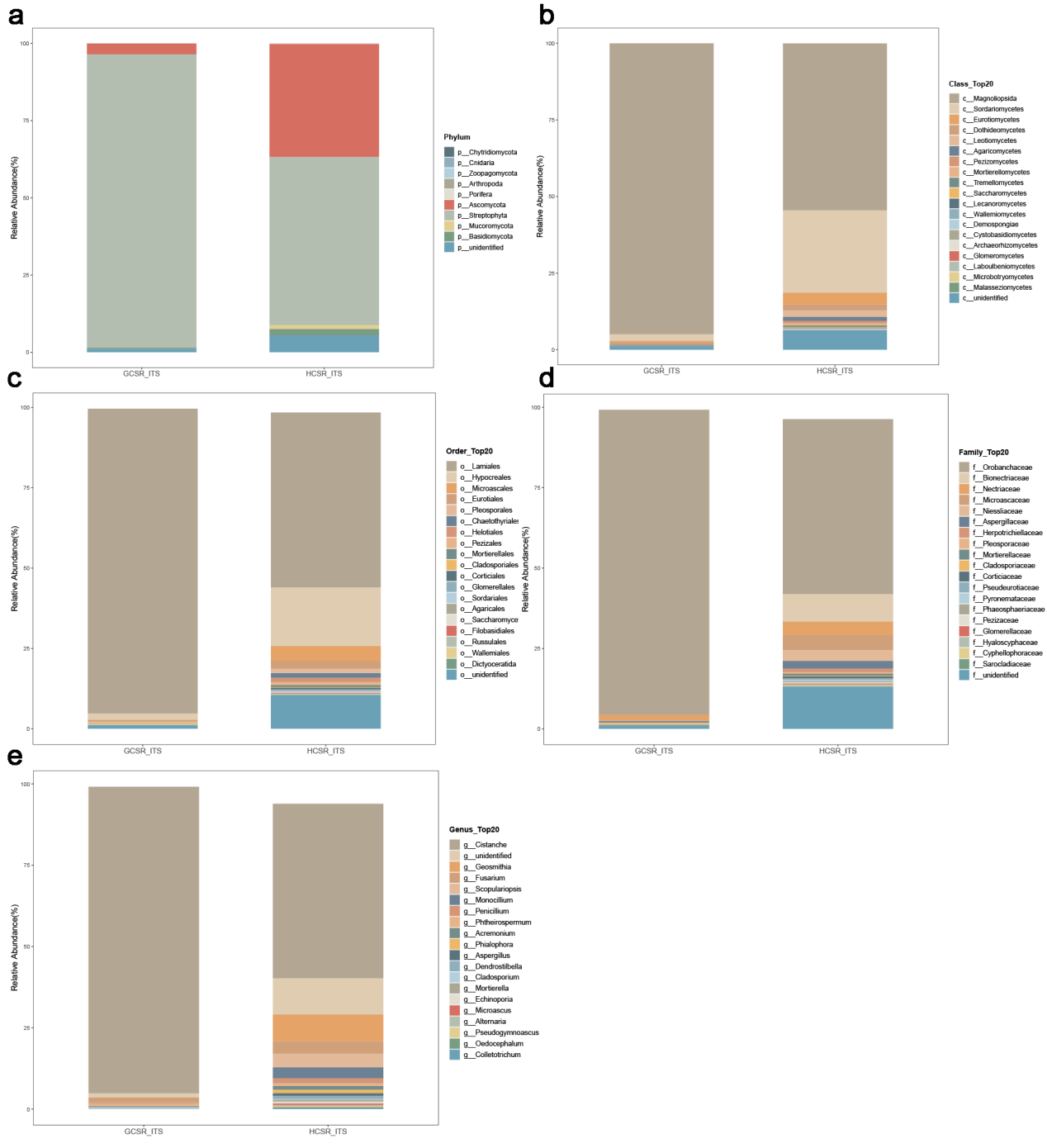


**Supplementary Figure 5.** Distribution of fungal species at different classification levels by groups. Selected fungal species with an abundance top 20 are displayed in the figure and combined other fungal species into vacancy; “unidentified” represents the fungal species without taxonomic annotation. Different fungal communities are distinguished by different color combinations. (a) Relative abundance of fungi at the phylum level. (b) Relative abundance fungi at the class level. (c) Relative abundance fungi at the order level. (d) Relative abundance fungi at the family level. (e) Relative abundance fungi at the genus level.


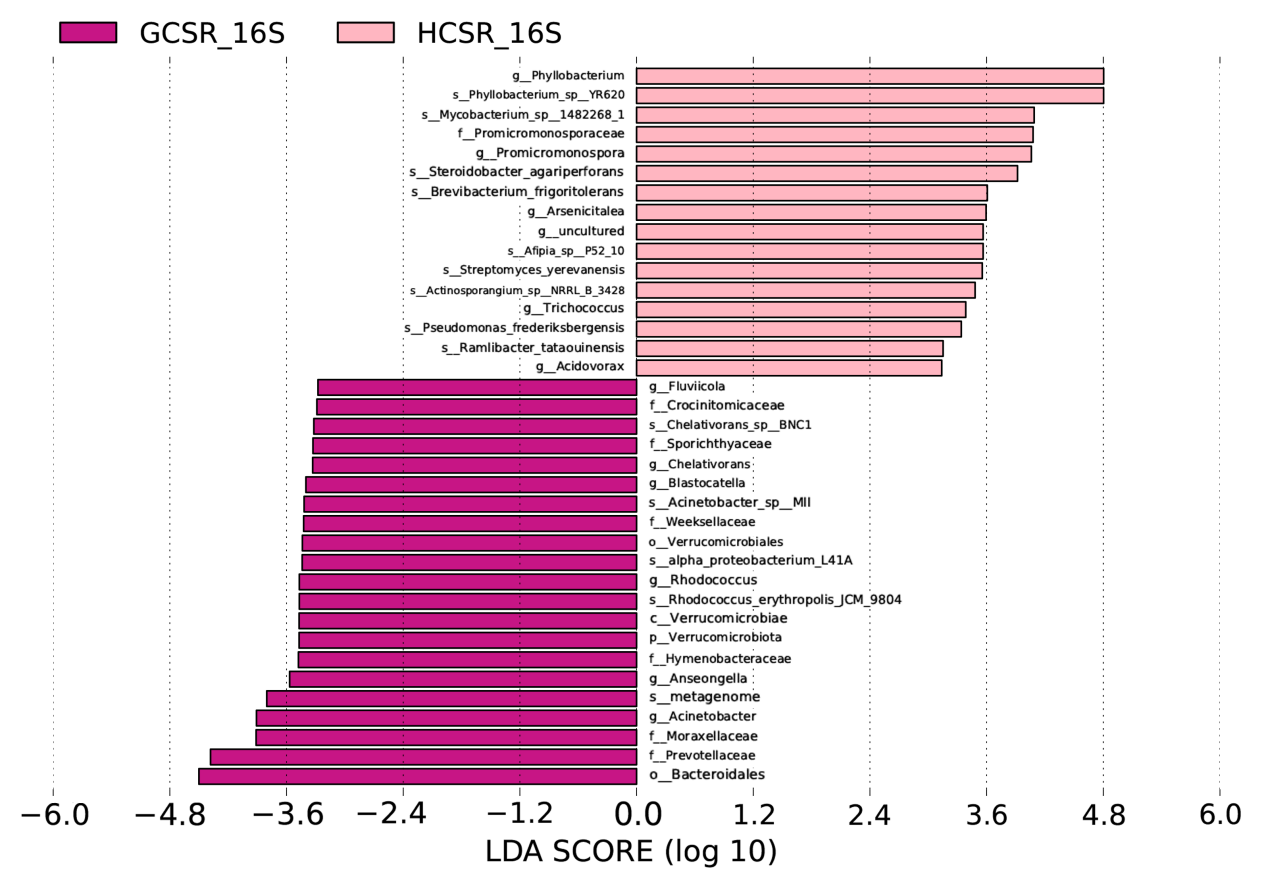


**Supplementary Figure 6.** The bacterial taxa with their LDA scores. Based on LEfSe results, the taxa were ranked according to their LDA scores.


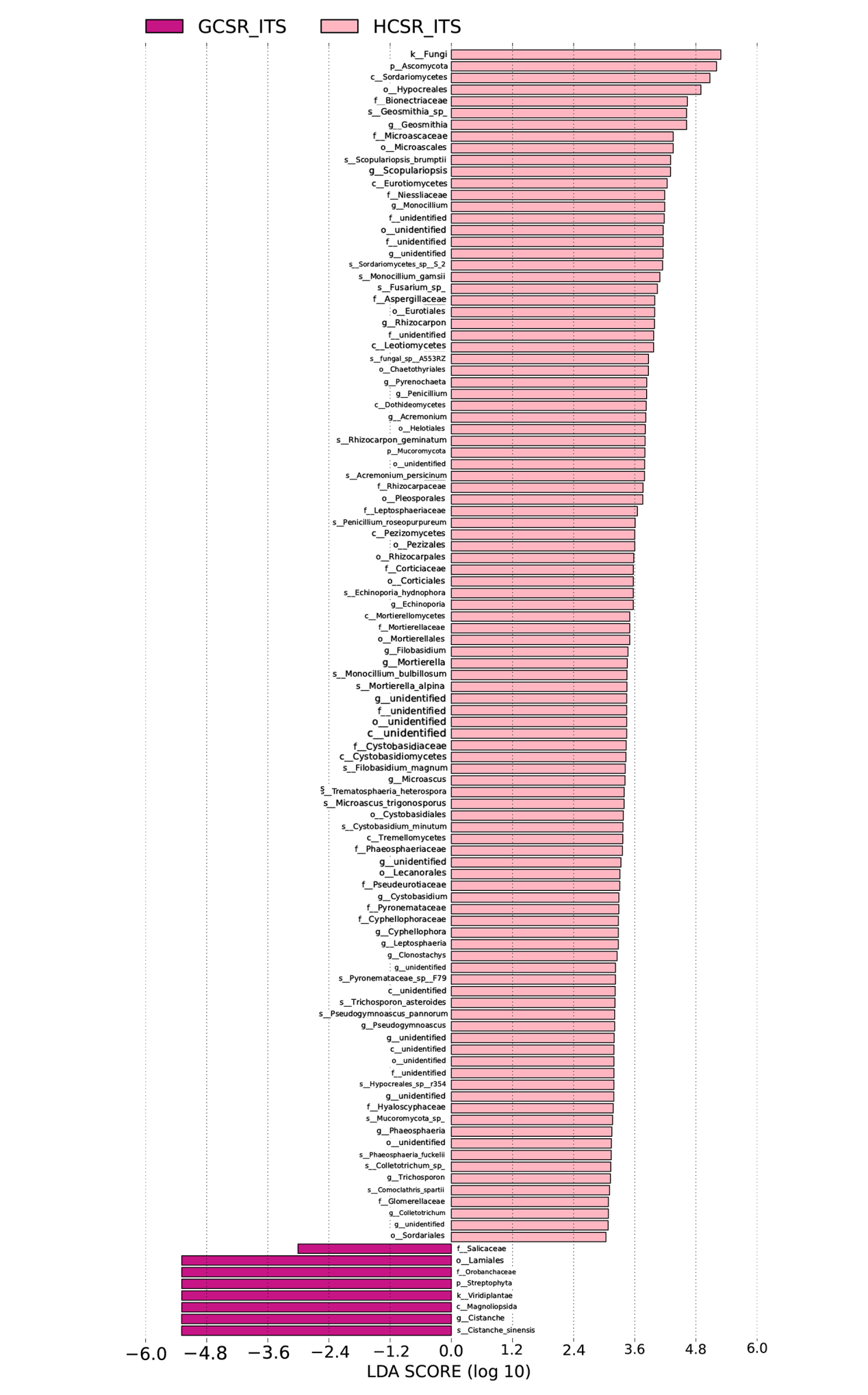


**Supplementary Figure 7.** The fungal taxa with their LDA scores. Based on LEfSe results, the taxa were ranked according to their LDA scores.
